# Survival characteristics and transcriptome profiling reveal the adaptive response of the *Brucella melitensis* 16M biofilm to osmotic stress

**DOI:** 10.3389/fmicb.2022.968592

**Published:** 2022-08-17

**Authors:** Jia Guo, Jiale Zhu, Tianyi Zhao, Zhihua Sun, Shengnan Song, Yu Zhang, Dexin Zhu, Shuzhu Cao, Xingmei Deng, Yingjin Chai, Yongxue Sun, Suleimenov Maratbek, Chuangfu Chen, Liangbo Liu, Hui Zhang

**Affiliations:** ^1^State International Joint Research Center for Animal Health Breeding, College of Animal Science and Technology, Shihezi University, Shihezi, China; ^2^Collaborative Innovation Center for Sheep Healthy Farming and Zoonotic Disease Control, College of Veterinary Medicine, South China Agricultural University, Guangzhou, China; ^3^College of Veterinary, National Agricultural University of Kazakhstan, Nur-Sultan, Kazakhstan

**Keywords:** transcriptome, *Brucella melitensis* 16M, ABC transporter, betaine, biofilm, osmotic stress

## Abstract

*Brucella* can inhabit hostile environments, including osmotic stress. How *Brucella* responds collectively to osmotic stress is largely unexplored, particularly in spatially structured communities such as a biofilm. To gain insight into this growth mode, we set out to characterize the *Brucella melitensis* 16M biofilm, describe its phenotype, and carry out a comparative transcriptomic analysis between biofilms under osmotic stress and control conditions. We determined that the bacteria challenged with 1.5 M NaCl had a reduced ability to aggregate and form clumps and develop a biofilm; however, the salt stress promoted the release of the outer membrane vesicles from the biofilm. Together with the genotypical response to osmotic stress, we identified 279 differentially expressed genes in *B. melitensis* 16M grown under osmotic conditions compared with control conditions; 69 genes were upregulated and 210 downregulated. Under osmotic stress, the main changed genes of biofilm were predicted to be involved in flagellar assembly, cell envelope, translation, small RNA regulation, transport and binding proteins, and energy metabolism. In addition, the ABC transporter was enriched in the Gene Ontology (GO) and Kyoto Encyclopedia of Genes and Genomes (KEGG) pathways. We highlight 12 essential ABC transporter genes associated with a bacterial response to osmotic stress at the biofilm stage, including one specific locus, BME_RS12880, mediating betaine accumulation in biofilms to eliminate osmotic stress. The current study results can help researchers gain insights into *B. melitensis* 16M biofilm adaptation to osmotic stress and provide information for developing intervention strategies to control *Brucella*.

## Introduction

Human brucellosis, caused by *Brucella* spp., is a zoonosis ravaging the Mediterranean region, Middle East, and Central Asia (Rossetti et al., 2017), causing severe health problems in humans and economic losses (Rossetti et al., 2017). *Brucella* has a variety of routes of infection, allowing it to survive within or out of mammalian hosts for a long period of time, even under hostile conditions, including nutrient limitation (Roop et al., 2003), phagosomal acidification (Porte et al., 1999), reactive oxygen damage (Jiang et al., 1993). A variety of *Brucella* genes have been implicated in the biology of *Brucella* infection, including those that affect multiple stress response pathways (Roop et al., 2009). Several recent studies have indicated that the general stress response mechanisms contribute to the adaptation of various chemical stressors *in vitro* and the establishment of chronic infection *in vivo* (Kim et al., 2013, 2014). However, there is relatively little information available about the mechanisms used by *Brucella* to adapt to stresses encountered in the environment. In addition, viable brucellae are able to persist in the environment, and periods of survival in soil, manure and water have been determined, reaching up to 180, 240, and 150 days, respectively (Crawford et al., 1990), research into this organism’s control is necessary.

Organisms are often exposed to abiotic stress factors such as radiation of ultraviolet (Gasch et al., 1998), fluctuations of temperature (Abu Bakar et al., 2020), osmotic imbalance (Paul, 2013), and damage of oxidative (da Cruz Nizer et al., 2021) during their lifetimes, *Brucella melitensis* can adhere to and stay on inert surfaces for long periods outside the host (Spinkal, 1956). Furthermore, *B. melitensis* has been reported to live for up to 90 days in ripened brine cheese (Galbraith et al., 1969; Clasessens and Ring, 1996), indicating a role of desiccation or osmotic stress tolerance in its persistence. The gaseous phase of a drying or dry environment with osmotic exposes bacteria to lower water activity than inside the cell, reducing water in the cells and triggering osmotic stress (Potts, 1994). Due to the disappearance of water and increased cellular exposure to the atmosphere, matric stress is characterized by osmotic and oxidative stress elements (Billi and Potts, 2002).

Physiological changes caused by severe salt stress on several bacteria have been pleiotropic. In *Bacillus subtilis*, the composition of the cytoplasmic membrane and cell wall are adjusted, and the aggregation capability of the cells is largely damaged (Steil et al., 2003). Moreover, salt stress alters the cell envelope composition of *Rhizobacteria*, causing changes in proteins, periplasmic glucans, capsular lipopolysaccharides (Altabe et al., 1994), and the composition of the cell envelopes, which play an important role in adaptation of osmotic stress (López et al., 2000). Under stressful conditions, certain bacteria like *Pseudomonas* produce exopolysaccharides (EPS), which enhance water retention and regulate the diffusion of carbon sources in the microbial environment to protect microbial from hydric stress and fluctuations in water potential (Sandhya et al., 2009). The development of biofilms associated with EPS was an essential process in the evolution of microbial cells, protecting the embedded cells against environmental insults, including mechanical shear, predation, invasion, and antibiotics (Flemming and Wingender, 2010; Dragoš and Kovács, 2017). Notably, stress tolerance to desiccation and osmosis in bacteria can be enhanced by biofilms under a variety of abiotic surfaces to meet certain specific requirements for microorganisms (Chowdhury et al., 2007; Almirón et al., 2013). Based on the above viewpoints, the protective role of the biofilm or EPS against environmental stresses is well known, as described for a *Nostoc commune* (Tamaru et al., 2005). However, the genetic factors and mechanisms that enable *Brucella* to adapt to osmotic stress in the biofilm stage remain understudied. Taking into consideration of the chronicity and epidemiology of *Brucella*, the characteristics of *Brucella* under these stress factors must be defined at the genetic level to support the development of eradication programs.

The objective of this research was to identify novel genetic factors contributing to osmotic tolerance in the *B. melitensis* 16M biofilm. In this study, we performed a transcriptome analysis of *B. melitensis* 16M biofilm under 1.5 M NaCl and control conditions and revealed that the osmotic stress tolerance of the *B. melitensis* 16M biofilm is a complex process involving flagellar assembly, cell envelope, translation, sRNA regulation, transport and binding proteins, and energy metabolism. Moreover, our data indicate that the ABC transporter, BME_RS12880, a glycine betaine target, contributes directly to the development of osmotic tolerance in the *B. melitensis* 16M biofilm. This research will provide insights into the correlation between osmotic stress and biofilm formation in *B. melitensis* 16M.

## Materials and methods

### Bacterial strains and growth conditions

The Beijing Institute of Disease Prevention and Control (Beijing, China) provided the strain of *B. melitensis* 16M strain used in the present study. All *B. melitensis* 16M strains were grown on Brucella agar (BD Difco, New Jersey, United States) for 2 days at 37°C and 5% CO_2_ (vol/vol) and in Brucella broth (BD Difco, New Jersey, United States) at 37°C with shaking until cultures reached an optical density at 600 nm (OD_600nm_) of 0.6 for biofilm culture. *Brucella melitensis* 16M-green fluorescent protein (GFP) was provided by Xinjiang Center for Disease Control and Prevention and was grown in Brucella broth with 30 μg/mL chloramphenicol to maintain the plasmid. All experiments with *Brucella* strains were performed in a biosafety level 3 facility.

### Biomass assay

A 2 ml (1.5 × 10^9^ CFU/ml) of *B. melitensis* 16M were seeded into Brucella broth in borosilicate tubes for 72 h. The planktonic phase in the borosilicate tubes was removed with phosphate-buffered saline (PBS), and the aggregates were passed over a 50-μm filter. Then, 250 μL 0.1% filtered crystal violet (CV) staining solution was added to filtrates and incubated for 15 min and washed three times with PBS and then fully dried in an incubator. Next, 200 μL 33% glacial acetic acid solution was added to each tube, and the borosilicate tubes were placed on a shaker to disperse the dye solution uniformly before the OD_550nm_ was measured.

### Flow cytometry analysis of bacterial viability in aggregates

Flow cytometry assay is emerging as an alternative rapid method for microbial detection, enumeration, and population profiling (Berney et al., 2007; Mudroňová, 2015; Pane et al., 2018; Vanhauteghem et al., 2019; Michelutti et al., 2020). And bacterial viability was assessed using the LIVE/DEAD BacLight™ kit (Thermo Fisher Scientific, United States) as described by the manufacturer. This bacterial viability kit is widely used in flow cytometry and consists of two nucleic acid stains: green fluorescent syto9 is cell-per-meable and freely enters all tested bacteria, either live or dead, while red fluorescent propidium iodide (PI) can only enter membrane-comprised cells (Berney et al., 2007). The method for determining bacterial viability (live/dead) by flow cytometry was based on previously published methods (Pu and Rowe-Magnus, 2018) with some modifications. 2 ml (1.5 × 10^9^ CFU/ml) of *B. melitensis* 16M were incubated for 72 h in Brucella broth under 1.5 M NaCl and control conditions, respectively. The aggregates were passed over a 50-μm filter and collected by centrifugation at 3,000 × *g* for 5 min and washed twice with PBS buffer solution, then 50 μL of Syto9/Pi fluorescent staining solution (Thermo Fisher Scientific, United States) was dropped into the filtrate and protected from light for 15 min, and flow cytometry of the filtrates was performed on a FACSCalibur flow cytometer (BD, United States) equipped with a blue and red laser (λex = 488 nm, 633 nm), bands passfilter measuring green and red fluorescence. The analyses were performed on biological triplicates and the data was analyzed using FlowJo X (Tree Star).

### Biofilm culture and confocal laser scanning microscope observation

Biofilms were cultured using 24-well plates following the previous descriptions and with some modifications (Almirón et al., 2013; Tang et al., 2021). Brucella broth was added to 24-well cell culture plates. Then a clean coverslip (8 mm × 8 mm, adhesion carrier) sterilized by autoclaving at 121°C for 15 min was placed in each well. The *B. melitensis* 16M-GFP suspension was inoculated at 2 ml/well (1.5 × 10^9^ CFU/ml) on the coverslip. Multilayered, dimensional biofilm under osmotic stress conditions was produced by inoculating bacteria onto the 3-dimensional nanofibrous scaffolds (Celevate, Sweden) placed in the 24-well plates. The plates were placed under UV light for 45 min as described by Okaro et al. (2019). Next, 1.5 M NaCl was added to induce osmotic stress. The culture plate was placed at 37^°^C with 5% CO_2_, the cells were incubated for 20 days with the culture medium changed every 10 days. The coverslips or scaffolds were removed and washed gently three times with PBS and then fixed immediately with 2.5% glutaraldehyde for 3–5 h at 4°C. These procedures were conducted to protect biofilms from falling off the abiotic surfaces (i.e., coverslips and scaffolds). The biofilm formed by *Brucella*-GFP was observed under confocal laser scanning microscopy (CLSM), confocal images of biofilm were then analyzed using NIS-Elements Viewer software 4.20 software (Nikon, Inc., Japan). The software reconstructed the two-dimensional intensity of fluorescence of all scanned layers into a three-dimensional volume stack at each cycle of scanning, and quantitative analysis of the images of the biofilms was performed using the Comstat2 program (Poudyal and Sauer, 2018). The biofilm biomass was also detected using the crystal violet staining method described above.

### Isolation and observation of outer membrane vesicles from *Brucella melitensis* 16M biofilm

The biofilms were inoculated and grown as described above. *Brucella melitensis* 16M biofilms were grown at 37°C for 20 days, then harvested with a cell scraper and immersed in 0.85% saline. A vortexing or homogenization step was used to liberate the outer membrane vesicles (OMVs) from the biofilm. After removing the biofilm, the saline solution was vortexed for 3 min, and then the cells were centrifuged for 20 min at 120,000 × *g*. After that, the supernatant is retained, and pellets were resuspended three times in 0.85% saline. We centrifuged the samples at 160,000 × *g* for 10 min and filtered the supernatant using a 0.45-micron filter to remove residual cells and cell debris. The OMVs were pelleted at 150,000 × *g* and 4°C for 75 min in a Thermo Scientific S50-A fixed angle rotor. The pellet was resuspended in MV buffer (50 mM Tris, 5 mM NaCl, 1 mM MgSO_4_, pH 7.4), sterilized with a 0.22-micron filter, and centrifuged again for 75 min at 150,000 × *g* at 4°C. After a second centrifugation step, the pellets were resuspended in 1 ml buffer, and carefully layered on a centrifuge tube containing a sucrose gradient ranging from 10 to 50%. The gradient was centrifuged at 120,000 × *g* for 5 h at 4°C in a swinging bucket rotor. The OMV band obtained between 20 and 30% sucrose was collected using a long syringe, and the collected OMVs were concentrated by centrifuging at 150,000 × *g* for 3 h. The pellet was resuspended in 500 mL of PBS buffer and filtered through a 0.22-micron filter. Total protein concentration was determined using the PIERCE-BCA (Thermo Fisher Scientific, United States) reagents, following the manufacturer’s recommendations. OMV samples were stored at –80°C until further use. In addition, the OMV was transferred to a copper net, placed in 2.5% glutaraldehyde solution, and fixed for 12 h. The OMVs were incubated in a phosphotungstic acid buffer of pH 7.2 for 30 s, dried, and observed under a HT7700 transmission electron microscope (TEM; Hitachi, Japan).

### Quantitative analysis of outer membrane vesicles isolated from biofilms

The purified OMV were quantified using two methods: the modified Lowry Protein Assay Kit (Thermo Fisher Scientific, United States), performed according to the manufacturer’s instructions, and nanoparticle tracking analysis (NTA) (Markwell et al., 1978; Cooke et al., 2020). For NTA, diluted pure OMVs (0.1 mg/mL) were loaded into the NTA chamber, and the particle size was recorded for 60 s at a laser wavelength of 488 nm using the ZetaView^®^ particle matrix analyzer (Particle Metrix, Germany).

### RNA-seq and data analysis

Biofilms of *Brucella* were grown in 24-well plates as described above. After 20 days of incubation, medium containing unattached planktonic bacteria were removed and the cells were washed twice with PBS. The attached bacteria, representing the biofilm fraction, were washed twice with PBS to remove any remaining planktonic cells. Attached cells were scrapped off the plate using a cell scraper. Biofilm fractions were subject to total RNA extraction using the TRIzol Max bacterial enhancement kit (Ambion, Life Technology, Carlsbad, CA, United States) as described by the manufacturer. RNA was further purified and concentrated using an RNeasy kit (Qiagen, China). rRNA was removed using the RiboZero magnetic kit (Illumina, United States). Sequencing libraries were generated using NEBNext Ultra Directional RNA library prep kit (NEB, United States) for Illumina. cDNA library quality and amount were verified using the Agilent Bioanalyzer 2100 system (Agilent Technologies, CA, United States) and then sequenced using Illumina NextSeq Mid-Output (Novogene Co., Ltd., Beijing). We downloaded the reference genome sequence from the National Center for Biotechnology Information (NCBI) database. The raw sequencing reads were cleaned by removing low-quality reads, reads containing poly-N sequences, and adaptor sequences. HISAT40 was used to align the reads to the reference genome (GCF_000007125.1). The expression value was measured in reads per kilobase per million mapped reads (RPKM). Then, the high-quality clean reads were compared with the specified reference genome by using Bowtie software. The Padj ≤ 0.05 and the absolute value of log_2_ ratio ≥ 2 were used to identify DEGs. The Gene Ontology (GO) and Kyoto Encyclopedia of Genes and Genomes (KEGG) databases were used to analyze the pathways.

### Quantitative real-time PCR

To validate the data generated from the RNA-seq experiment, 12 genes of the ABC transport system were selected for further analysis *via* quantitative real-time PCR (qRT-PCR). After 20 days of culturing *B. melitensis* 16M strains, the biofilm developed was washed three times with PBS and RNA molecules were directly extracted from the biofilm. The processed sample was poured into an agate mortar that had been sterilized in advance, and liquid nitrogen was added to quickly grind the sample into a thick liquid. An RNA extraction kit was used for RNA isolation (Takara, United States). The primers are listed in Supplementary Table 1. The qRT-PCR conditions consisted of 5 min at 95°C for pre-incubation, followed by 40 cycles at amplification (95°C for 30 s, 58°C for 30 s, and 72°C for 30 s). The samples were evaluated in triplicate and amplified in a 20-μL reaction containing 2 × SYBR Premix Ex Taq II (Takara, United States). qRT-PCR was carried out with a Roche Light Cycler 480 II system (Basel, Switzerland), with 100 nM each primer and 1 μg cDNA target. All assays were performed three times.

### Construction of bacterial mutants and complementary strains

Primer 5.0 software was used to design the homologous primers for the gene of interest based on the gene sequence of the international standard strain of *B. melitensis* (NC_003317.1, NC_003318.1) published in the GenBank. The BME_RS12880 mutants were constructed as previously described (Li et al., 2017). The primers used to construct the mutant are shown in Supplementary Table 2. Briefly, the primer pair Kan-F and Kan-R was synthesized to include restriction enzyme sites for *KpnI* at the 5’ end of Kan-F and *PstI* at the 5’ end to Kan-R. The *Kan* gene was amplified from pBBR1MCS4 with these primers and the PCR products were cloned into the pMD19-T vector (Takara, Shiga, Japan). The positive clones were sequenced and then subcloned into pUC19 *via* the *KpnI* and *PstI* sites to generate the pUC19K plasmid. The upstream and downstream homologous fragments of the target gene were amplified and cloned into pUC19K to generate a suicide plasmid. Electrocompetent *B. melitensis* 16M cells were prepared and transformed with the pUC19K-BME_RS12880 plasmids. Transformants were selected in the presence of 100 μg/mL ampicillin and 100 μg/mL kanamycin. *Trans*-complementation of the mutants was conducted with the pBBR1MCS4 plasmid that included the native gene (Elzer et al., 1995) using the same transformation method in the mutant strains. The primers used to complement are shown in Supplementary Table 2.

### Prediction and analysis of protein structure and function

The functional domains of proteins were predicted using the NCBI CDD^1^ software. The String software was used to analyze the Protein-Protein Interaction (PPI) network map of the ABC transporter–*Brucella*-potential target genes and corresponding signal pathways.^2^

### Molecular docking

The 3D structure of the target BME_RS12880 was generated from the Phyre2 Server.^3^ The structure of betaine (compound CID: 247) was downloaded from PubChem.^4^ The structures of the compound and target BME_RS12880 for the betaine were optimized by conducting energy minimization, removing water molecules, and incorporating non-polar hydrogens using ChemBio3D Ultra7.0 (Buntrock, 2002) and the Autodock Tool 1.5.6 (Morris et al., 2009). Blind docking was subsequently performed using the Autodock Tool 1.5.6 software, and 10 predictions were performed. The docking model with the lowest binding energy score was selected and visualized using PyMOL 1.7 (Rigsby and Parker, 2016) to clarify the binding sites and interactions between the key targets and compounds.

### Cells culture and infection

RAW 264.7 cell lines were purchased from Cell Resource Center (Shanghai, China) and cultured in Dulbecco’s modified Eagle’s medium (DMEM) supplemented with 10% fetal bovine serum (Gibco, United States) under 37°C and 5% CO_2_ conditions. Prior to infection, RAW 264.7 were seeded to properculture plates at a density of 10^5^ cells/mL in complete culture medium without penicillin and streptomycin, and cells were infected with wt, ΔBME_RS12880, and ΔBME_RS12880-C strains at a multiplicity of infection (MOI) of 50. Culture plates were centrifuged at 350 × *g* for 5 min at room temperature and incubated at 37°C for 60 min. After washing twice with PBS, the infected cells were incubated for an additional 45 min in the presence of 50 mg/mL of gentamicin to kill extracellular bacteria. Then, the cultures were placed in fresh DMEM containing 25 mg/mL of gentamicin and incubated at 37°C. At 0, 4, 8, 12, 24, and 48 h post-infection, the supernatant was discarded and cells were washed three times with PBS and lyzed by PBS containing 0.1% (v/v) Triton X-100. Ten-fold serial dilutions of the lysates containing live bacteria were performed and enumerated the CFU by plating on Brucella agar plates. All assays were performed in triplicate and repeated at least three times.

### Statistical analyses

Statistical analyses were performed using GraphPad Prism software. Data from multiple groups were analyzed by one-way ANOVA with Dunnett’s multiple comparison test, whereas the unpaired Student’s *t*-test was used to compare two groups. A probability (*P*) value ≤ 0.05 was considered significant.

## Results

### Osmotic stress inhibits the large aggregates of *Brucella melitensis* 16M

**FIGURE 1:**
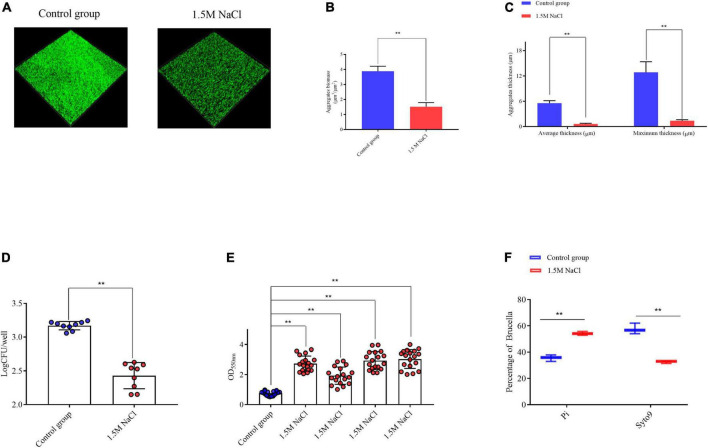
Analysis of *B. melitensis* 16M aggregation ability in 1.5 M NaCl and control conditions. **(A)** Live confocal imaging of stacks of *B. melitensis* 16M-GFP aggregates grown for 72 h in Brucella broth medium with or without 1.5 M NaCl. Scale bars = 20 μm. Confocal images were subjected to quantitative analysis using the Comstat2 program to determine the aggregates biomass **(B)** and the average and maximum aggregates thickness **(C)**. **(D)** Survival of bacteria in aggregates grown with or without 1.5 M NaCl treatment. **(E)** The biomass of aggregates was quantified with 0.1% CV. **(F)** Flow cytometry analysis of the live/dead status of bacteria in the biofilm filtrates. Error bars represent standard error (*n* ≥ 3). **P ≤ 0.01, unpaired Student’s *t*-test.

After 72 h of static culture, CLSM showed that the *B. melitensis* 16M exhibited a clumping phenotype characterized by large aggregates and settled on the coverslip surface, as evident by the biomass, which was a average of 3.8 μm^3^/μm^2^; average and maximum aggregates thickness, which were 5.5 and 12.3 μm, respectively (Figures 1A–C). This aggregation phenotype was disrupted in *B. melitensis* 16M treated with 1.5 M NaCl, average biomass was characted as 1.4 μm^3^/μm^2^, average and maximum aggregates thickness, which were 0.6 and 1.4 μm, respectively (Figures 1A–C). And also reduced bacterial viability of aggregates (Figure 1D). To clarify whether the bacterial growth defects were due to the osmotic stress produced by the 1.5 M NaCl, we found that high concentrations of KCl, sucrose and dextran also reduced the viability of *Brucella* (Supplementary Figures 1A,B). Biomass counts in 50-μm filtrates relative to the aggregates of the culture were measured for the control group and osmotic stress conditions. The biomass of the *B. melitensis* 16M aggregates in the culture was very low compared with the aggregates of the culture with added 1.5 M NaCl (Figure 1E). This indicates that most biomass aggregates in the culture were recovered in the 50-μm filtrate, in contrast to the control group. The filtrates of the treated *B. melitensis* 16M showed smaller clumps and only about half of them were removed following filtration. We also performed live/dead staining with the LIVE/DEAD bacterial viability kit to assess whether the aggregate cells of the filtrate were alive or dead. FACS analysis of the filtrates was used to analyze the aggregates’ active distribution in the filtrates accurately. More live bacteria was detected in the filtered aggregates in the control group than in the treatment (Figure 1F). The result shows that the aggregates in the control group were too large to traverse the filter. The osmotic stress weakened the growth and development states of the large aggregates, suggesting that osmotic stress may affect the aggregation ability of the *B. melitensis* 16M biofilm in the early stage.

### Phenotype changes of *Brucella melitensis* 16M biofilm under osmotic stress

**FIGURE 2:**
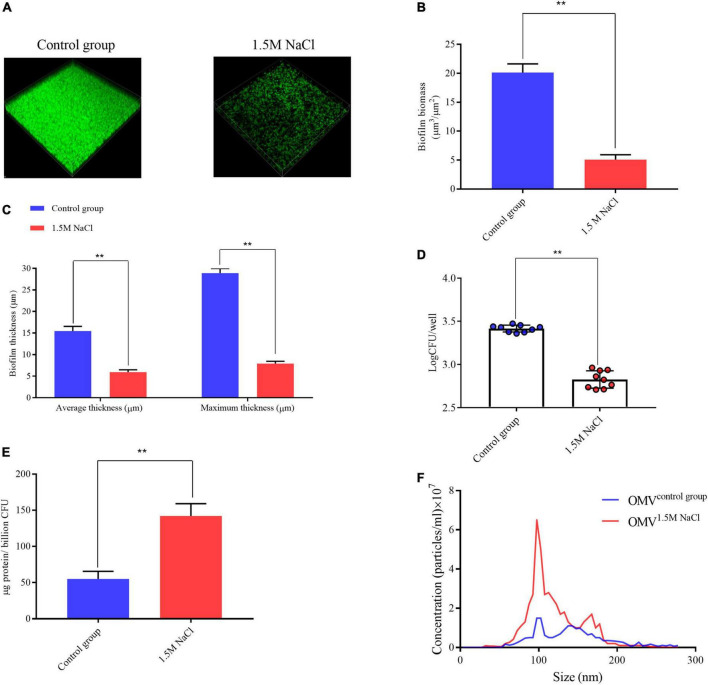
*Brucella melitensis* 16M biofilm under 1.5 M NaCl and control conditions. **(A)** Live confocal imaging of stacks of *B. melitensis* 16M-GFP biofilms grown for 20 days in Brucella broth medium with or without 1.5 M NaCl. Scale bars = 20 μm. Confocal images were subjected to quantitative analysis using the Comstat2 program to determine the biofilm biomass **(B)** and the average and maximum biofilm thickness **(C)**. **(D)** Survival of bacteria in biofilms grown with or without 1.5 M NaCl treatment. Mean values in each treatment group were statistically compared using a two-tailed unpaired *t*-test. Data are representative of at least five biological replicates, with each point representing a biological replicate. **(E)** Purified OMV were quantified by the modified Lowry assay and normalized to micrograms protein per billion CFU. **(F)** Nanoparticle tracking analysis measurement of OMV preparation (0.1 mg protein/mL) showing the sizes and total number of OMV per mL. Error bars represent standard error (*n* ≥ 3). **P ≤ 0.01, unpaired Student’s *t*-test.

We initiated our work by evaluating the physiology of *B. melitensis* 16M biofilm under microaerobic conditions (Almirón et al., 2013) and investigated the response of the *B. melitensis* 16M biofilm in a 1.5 M NaCl solution. Biofilms were grown in Brucella broth media in the presence of 1.5 M NaCl under microaerobic conditions. The biofilms were visualized by CLSM. After 20 days of growth in the Brucella broth, the control group formed bacterial biomass that homogeneously covered the surface, consistent with a well-developed biofilm, with a average biomass of 19.6 μm^3^/μm^2^, average and maximum thickness, which were 15.4 and 28.8 μm (Figures 2A–C). Moreover, the biofilms formed under the 1.5 M NaCl appeared to be composed of fourfold less biomass than the biofilms formed under control conditions (Figures 2A–C), we obtained consistent results with crystal violet staining and bacterial plate count (Figure 2D and Supplementary Figures 2A,B). However, we isolated more biofilm-derived OMVs under osmotic conditions and quantified using two independent techniques: OMV protein quantification and NTA. Modified Lowry assays showed that the highest protein levels were detected in OMV preparations harvested in the biofilm under 1.5 M NaCl contions (Figure 2E). Protein concentrations in OMV pellets were normalized per billion CFU. Quantification *via* NTA (which counts OMV particles directly) demonstrated that the size of the outer membrane vesicles under the two conditions is concentrated around 100 nm (Figure 2F), and there is almost no change in size, which is basically consistent with our morphological identification (Supplementary Figure 3A), but more outer membrane vesicles are isolated under the osmotic stress condition (Figure 2F and Supplementary Figure 3B). These results suggest that osmotic conditions alter the biological characteristics of *Brucella* biofilms.

### Phenotypes changes in osmotic stress of *Brucella melitensis* 16M are independent of a multilayered three-dimensional structure

After observing the *B. melitensis* 16M biofilm phenotypes under osmotic stress, we questioned whether the multilayered structure of the biofilm itself could withstand the damaging effects of this osmotic pressure. CLSM was employed to examine images of the biofilm produced by *B. melitensis* 16M grown on a 3-dimensional nanofibrous scaffold. CLSM analysis showed that the control group exhibited intact adhesion and aggregation compared with the hyperosmotic stress treated samples, as seen in the top row images. The control group produced more biomass (Supplementary Figures 4A–D) and maintained a more active bacterial state (Supplementary Figure 4E). Our results suggest that multilayer biofilms do not compensate for osmotic stress damage to biofilms.

### Differential gene expression between *B. melitensis* 16M grown-biofilm under osmotic stress and control conditions

**FIGURE 3:**
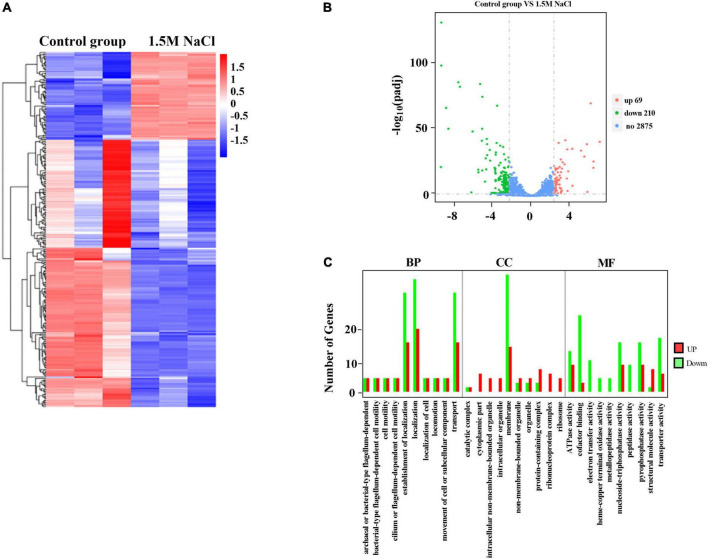
Expression of differentially expressed genes (DEGs) between the *B. melitensis* 16M biofilm under 1.5 M NaCl and control conditions. **(A)** The heatmap shows the expression levels of DEGs between the *B. melitensis* 16M biofilm under 1.5 M NaCl and control conditions. **(B)** Volcano plot of expressed genes between *B. melitensis* 16M biofilm under 1.5 M NaCl and control conditions. The red, green, and blue denote upregulated, downregulated, and non-regulated genes, respectively, in the *B. melitensis* 16M biofilm under 1.5 M NaCl compared with the control conditions based on the following criteria: absolute log_2_ (fold change) ≥ 2 and adjusted *P* ≤ 0.05. **(C)** GO terms analysis the number and function of differentially expressed genes (DEGs) between the *B. melitensis* 16M biofilm under 1.5 M NaCl and control conditions. BP, biological process; CC, cellular component; MF, molecular function.

**FIGURE 4:**
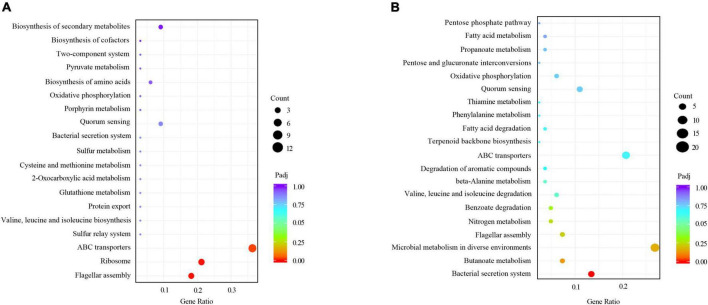
KEGG analyses of the differentially expressed genes (DEGs) between the *B. melitensis* 16M biofilm under 1.5 M NaCl and control conditions. The rich factor represents the ratio of **(A)** upregulated genes and **(B)** downregulated genes differentially expressed gene numbers annotated in this pathway term to all gene numbers annotated with this pathway term. A greater rich factor indicates a greater degree of pathway enrichment. The padj represents the corrected *P*-value and ranges from 0 to 1, and a lower value indicates greater pathway enrichment.

We employed a transcript profiling experiment using transcriptome sequencing (RNA-seq) to understand the *B. melitensis* 16M biofilm genes involved in responding to osmotic stress. The gene expression profiles of the *B. melitensis* 16M grown-biofilms under osmotic stress and control conditions were compared, and the gene expression levels were analyzed by Illumina HiSeq™ 2000. Based on their expression levels, cluster analysis was used to arrange the samples into groups to elucidate possible relationships among the samples (Figure 3A). Our comparative transcriptomic analysis revealed 279 differential DEGs (Padj ≤ 0.05, fold change ≥ 2), of which 69 genes were upregulated (Supplementary Table 3), and 210 genes were downregulated (Supplementary Table 4). The Volcano Plot is shown in Figure 3B. We performed a GO enrichment analysis to explore the biofilms’ biological processes in responding to osmotic stress. The establishment of localization, transport, membrane and cofactor binding were the dominant groups in all three DEG sets (Figure 3C). Based on KEGG pathway enrichment analysis, most upregulated genes involved flagellar assembly, ribosome, and ABC transporters (Figure 4A). The major downregulated genes were involved in the bacterial secretion system, butanoate metabolism and microbial metabolism (Figure 4B). In the GO and KEGG analysis, transport is the main pathway in responding to osmotic stress in biofilms (Figures 3C, 4A).

### Osmotic stress activated the ABC transport pathway in *B. melitensis* 16M biofilm

**FIGURE 5:**
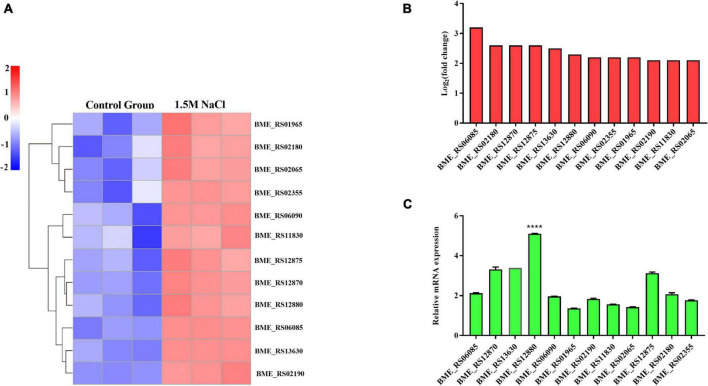
Differentially expressed genes (DEGs) were evaluated by quantitative reverse transcription PCR (qRT-PCR) assays between the *B. melitensis* 16M biofilm under 1.5 M NaCl and control conditions. **(A)** The heatmap shows the expression levels of 12 ABC transporters between the *B. melitensis* 16M biofilm under 1.5 M NaCl and control conditions. **(B**,**C)** The 12 ABC transporters expression levels were further detected by qRT-PCR. The results at each time point are expressed as the means ± standard deviations from at least three independent experiments. One-way ANOVA was used followed by Dunnett’s test to assess significance. ****P ≤ 0.0001.

Previous studies have indicated that the ABC transporters play a central role in metabolic and energy-producing pathways in bacteria that respond to osmotic stress (Tam and Saier, 1993; Warmbold et al., 2020). The gene expression changes related to ABC transporter pathways were assessed to explore whether this pathway can respond to osmotic stress in the *B. melitensis* 16M biofilm. Specifically, upregulated genes involved in the ABC transporter pathways included BME_RS06085, BME_RS12870, BME_RS13630, BME_RS12880, BME_RS1 2875, BME_RS01965, BME_RS02190, BME_RS06090, BME _RS02065, BME_RS02355, BME_RS02180, BME_RS11830. The expressions of these genes using qRT-PCR were in good agreement with the RNA-seq results (Figures 5A–C). This finding agrees with a previous report that some ABC transporters were induced by salt stress to adapt to osmotic stress. They are involved in the transportation of compatible solutes and have the most significant protective effect on growth. Notably, the BME_RS12880 gene was significantly elevated (Figure 5C). The BME_RS12880 encoded OpuA like protein is reported to be involved in osmotic regulation (Hoffmann et al., 2013). Recent work on the osmoregulated transporter OpuA from *Lactococcus lactis*, showed that this OpuA protein act as one of a few both osmosensor and osmoregulator (van der Heide and Poolman, 2000). Thus, we speculate that the BME_RS12880 gene is involved in the regulation of osmotic stress in *B. melitensis* 16M biofilm.

### BME_RS12880 gene involved in the osmotic stress response of the *B. melitensis* 16M biofilm

**FIGURE 6:**
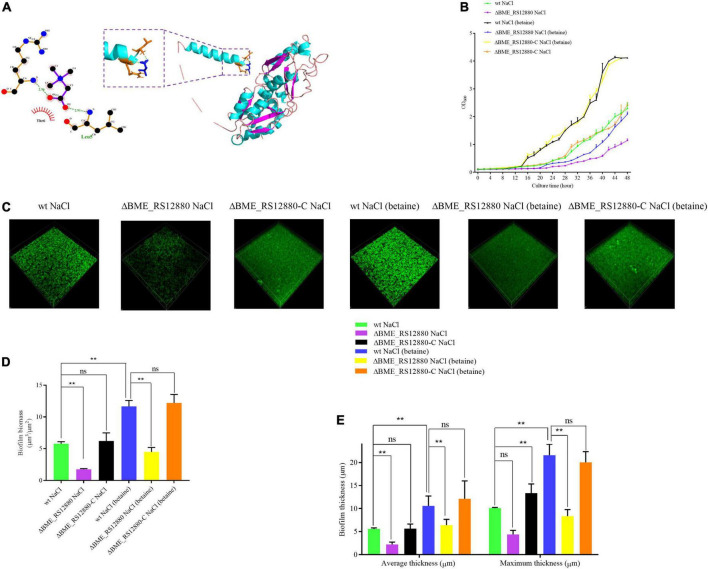
Functional analysis of ΔBME_RS12880 in response to osmotic stress. **(A)** Molecular model of BME_RS12880 binding to betaine. The light blue cartoon structure indicates the BME_RS12880, the navy blue structure indicates betaine, the structure consisting of light blue and brown represents the residues bound to betaine, and the green dashed lines indicate hydrogen bonds. **(B)** Growth of the wt, ΔBME_RS12880, and ΔBME_RS12880-C strain in Brucella broth under osmotic or supplemented with 1 mM betaine. Error bars represent the standard deviations of three independent experiments. **(C)** Live confocal imaging of stacks of ΔBME_RS12880 and ΔBME_RS12880-C strain biofilms grown for 20 days in Brucella broth medium under osmotic or supplemented with 1 mM betaine. Scale bars = 20 μm. Confocal images were subjected to quantitative analysis using the Comstat2 program to determine the biofilm biomass **(D)** and the average and maximum biofilm thickness **(E)**. All experiments were carried out at least in triplicate. Error bars represent standard error (*n* ≥ 3). One-way ANOVA was used followed by Dunnett’s test to assess significance. **P ≤ 0.01.

Based on the above results, we speculate that the BME_RS12880 gene may be involved in the osmotic stress response of the *B. melitensis* 16M biofilm. Comparing the BME_RS12880 protein sequences to models in the Conserved Domain Database (CDD) revealed high homology to components of an ABC transmembrane transport system (Supplementary Figure 5A). The most significant probability scores were for ABC transporters that import compatible solute molecules (glycine betaine) to mitigate the effects of osmotic stress. Protein-protein interaction (PPI) networks can better reflect the interactions between proteins. Through PPI pathway analysis, we predicted and analyzed the main known and potential proteins interacting with BME_RS12880. Identifying these proteins will help analyze the function and action network of BME_RS12880-interacting proteins. Combined with the transcriptome of the *B. melitensis* 16M biofilm, we found that the proteins encoded by the three genes BME_RS12880, *proV* and BME_RS12875 had potential interactions and increased transcription levels under osmotic stress (Supplementary Figure 5B and Supplementary Table 3). Thus BME_RS12880 may be involved in the osmotic stress response of the biofilm together with these genes. Therefore, the binding model of BME_RS12880 protein and betaine was predicted by molecular docking analysis. The results indicate that BME_RS12880 binds to betaine *via* two hydrogen bonds on its arginine residue (Figure 6A).

Thus, we assayed the growth of wt, ΔBME_RS12880, and ΔBME_RS12880-C strains under 1.5 M NaCl or added 1 mM betaine. Although wt and ΔBME_RS12880 strains had equivalent phenotypes under control conditions (Supplementary Figure 6), the ΔBME_RS12880 strain had a growth defect compared with the wt under 1.5 M NaCl, and complementation restore the wt phenotype (Figure 6B). Adding 1 mM betaine to the wt alleviates the inhibitory effects of osmotic stress for *Brucella* (Figure 6B). However, in ΔBME_RS12880, betaine did not effectively relieve the inhibition of osmotic pressure, and ΔBME_RS12880-C could effectively restore the growth inhibition caused by osmotic stress (Figure 6B). It also shows that betaine can be used as an osmoprotectant for *Brucella.* It can accumulate to high concentrations inside the cells by transport from the medium and thereby alleviate the inhibitory effects of osmotic stress. We also investigated the impact of the BME_RS128801 and betaine in the biofilm under 1.5 M NaCl. Our results suggest that BME_RS128801 is also involved in biofilm responses to osmotic stress, The average biomass of ΔBME_RS12880 biofilm under 1.5 M NaCl was characted as 1.76 μm^3^/μm^2^ and average and maximum aggregates thickness, which were 2.16 and 4.38 μm, respectively. Moreover, the biofilms formed by ΔBME_RS12880 strain appeared to be composed of threefold less biomass than the biofilms formed by wt strain under the 1.5 M NaCl, and ΔBME_RS12880-C restore the ΔBME_RS12880 phenotype (Figures 6C–E). While adding 1 mM glycine, it can effectively restore the damage of osmotic stress to the biofilm, as evident by the biomass, which was a average of 11.7 μm^3^/μm^2^ and average and maximum aggregates thickness, which were 10.6 and 21.2 μm, respectively, which appeared to be composed of twofold more biomass than the biofilms formed under 1.5 M NaCl without 1 mM glycine (Figures 6C–E). However, deletion of the ΔBME_RS12880 gene causes this compensatory effect of glycine (Figures 6C–E). We obtained consistent results with crystal violet staining (Supplementary Figure 7A). These results suggest that glycine can effectively counteract the damage of osmotic stress on *B. melitensis* 16M biofilm, and this process is mediated by the BME_RS12880 gene. To determine whether the osmotic stress-induced biomass reductions are caused by increased cell death, we quantified the survival of biofilm cells after 20 days of osmotic stress in a colony biofilm model. Compared with the wt, ΔBME_RS12880 biofilms had significantly reduced cell survival after treatment with 1.5 M NaCl or betaine, indicating ΔBME_RS12880 biofilm cells are more sensitive to 1.5 M NaCl-induced cell death (Supplementary Figure 7B).

In addition, we established a macrophage model of *Brucella* infection. For 8 h, we observed no difference in replication defects between the wt strain and the ΔBME_RS12880 strain. After 8 h, a significant growth defect was found (Supplementary Figure 8). It provides evidence that the deletion of ΔBME_RS12880 does affect the potential of *B. melitensis* 16M to infect and replicate under *in vitro* infection models. Based on the above results, we inferred that ΔBME_RS12880 affects the development of biofilms *via* a betaine transporter pathway under osmotic stress and may be involved in the intracellular survival of *Brucella*.

## Discussion

Throughout evolution, organisms have developed effective osmoadaptation mechanisms, including accumulating compatible solutes, ion transport, and energy production to adapt to osmotic stress (Sleator and Hill, 2002). In addition, under less favorable conditions, the biofilm plays an essential role in bacterial survival and growth as a result of stress regulation in response to changing environmental conditions (Venkatesan et al., 2015). It has been reported that *Brucella* could form a biofilm or aggregate as a response to desiccation or osmoadaptation (Tamaru et al., 2005). The molecular mechanisms by which *Brucella* biofilms resist osmotic stress remain largely uncharacterized. This study showed less bacterial aggregation and clumps, biofilm formation and enhanced secretion of OMVs from the biofilm under 1.5 M NaCl. We used RNA sequencing and comparative transcriptomic analysis between the biofilm cells under 1.5 M NaCl and those under the normal growth conditions to gain insights into the mechanisms behind the biofilm-related phenotypic changes. We identified distinct expression profiles between hyperosmotic stress and normal cells of the biofilm in *B. melitensis* 16M. A total of 279 genes were exclusively differentially expressed in *B. melitensis* 16M grown under 1.5 M NaCl conditions compared with the control. To further investigate the biological functions of these genes, the DAGs were mapped to the GO and KEGG pathways. The associated cellular processes discussed below could be classified into several main sections: flagellar assembly, cell envelope, sRNA regulation, transport and binding proteins, energy metabolism, and translation. We summarized the above analyses and formed a gene change model of *B. melitensis* 16M biofilm cell adaptation to osmotic condition. In particular, we identified an ABC transporter pathway gene in the transcriptional regulation pathway and showed that this ABC transporter BME_RS12880 played an important role in resisting hyperosmotic stress in the *B. melitensis* 16M biofilm. We provide new insights into the molecular mechanisms of osmotic adaptation in *Brucella*.

The significantly elevated of several flagellar transcripts is one fascinating finding in the biofilm challenged by osmotic stress. Notably, these were not for the entire flagellum but specific genes encoding the hook (*flgL*, *flgE*, *flgD*, *flgK*), flagellin (*fliC*), flagellar regulator (*flaF*, *flbT*), and flagellar biosynthetic protein (*fliQ*). This result suggests that the upregulation of flagellum-associated gene expression may be a valuable survival mechanism for cells experiencing biofilm formation under osmotic stress. The process of flagellum biosynthesis is energy-intensive (Macnab, 1996), in response to stress, bacterial cells would likely benefit from downregulating flagellum-associated gene expression and redirecting their energy to more critical metabolic functions. It has been suggested that flagella genes are turned off in mature biofilms or in some specific state of bacteria (Whiteley et al., 2001; Sauer et al., 2002; Guttenplan and Kearns, 2013).

Nevertheless, other studies have demonstrated that flagella genes are expressed by some microbes at every stage of biofilm development, not just during attachment and dispersion of biofilm (Serra et al., 2013; Kim et al., 2015). Flagellar biosynthesis genes have been identified in *Escherichia coli* biofilms. Around 20 flagellar genes are maintained throughout the development of biofilms and are not turned off (Domka et al., 2007), suggesting acting to cement and support the cells together and to the surface with other matrix components (Hung et al., 2013; Valentin et al., 2022). Genomic sequencing of isolated non-motile *Brucella* has detected the existence of non-functional flagellar genes. In contrast (Léonard et al., 2017), the analysis of genes encoding flagellar proteins in the motile strain revealed that all genes were fully functional (Soler-Lloréns et al., 2016), emphasizing that some genes may be expressed under environmental selection pressure and perform specific biological functions (DelVecchio et al., 2002; Wareth et al., 2017). An example of its adaptive functions includes the activation of flagellar genes in response to environmental changes or stress (Mirabella et al., 2013; Petersen et al., 2013) and evidence of the expression of *Brucella* flagellar proteins, which are involved in virulence (Fretin et al., 2005), infectivity (Fretin et al., 2005), cell growth, and biofilm formation (Coloma-Rivero et al., 2020). However, some papers describing the expression of the flagellar genes of *B. melitensis* agree that the flagellin gene expression is maximal at the logarithmic growth phase and disappears in the exponential phase (Fretin et al., 2005; Ferooz and Letesson, 2010). The mechanism by which flagellar genes respond to changes in osmotic pressure remains to be investigated.

The outer membrane (OM) of Gram-negative bacteria prevents nutrients and toxic molecules from entering (Hancock, 1997). It also uses protein channels, known as porins, within its OM to facilitate selective nutrient and molecule entry to promote cell growth (Xiao et al., 2016). We found that the gene encoding the OmpW protein (BME_RS02270) was downregulated in the biofilm under osmotic stress. Despite OmpW is a minor porin protein in Gram-negative bacteria, it has been implicated in bacterial responses to various antibiotics stresses. For instance, in *Vibrio cholerae*, the expression of OmpW was affected by a broad range of cultural conditions such as temperature, salinity, and availability of nutrients or oxygen. Consequently, it was considered to be involved in the stress adaption of the bacterium (Nandi et al., 2005). However, no defined physiological functions for OmpW have been reported in *Brucella*. The genes, BME_RS10345, BME_RS10370, *exbD*, *exbB*, genes encoding the lipoprotein, the outer membrane protein OmpA, TonB system transport protein ExbD, TonB-system energizer ExbB were also downregulated in the biofilm under osmotic stress. These genes have previously been associated with the envelope stability of Gram-negative bacteria, which is essential for bacterial physiology and survival in a constantly changing environment (Szczepaniak et al., 2020; Paulsson et al., 2021). Maintenance of this envelope stability is often achieved by coordinated regulation of different envelope crosslinks that involve (i) the covalent crosslinking of Braun’s lipoprotein (Lpp) in the outer membrane with the PG sacculus; (ii) the non-covalent interactions between the PG and OmpA, which is an outer-membrane porin; and (iii) the non-covalent interactions between the PG and the Tol–Pal (peptidoglycan-associated lipoprotein) complex (Schwechheimer and Kuehn, 2015). Strains with mutations in envelope components often have lytic phenotypes, making comparisons with wild-type bacteria challenging (Schwechheimer and Kuehn, 2015). Hypersensitivity to environmental pressure releases more outer membrane vesicles. The utility of OMVs in promoting envelope homeostasis and preventing toxicity appears to be supplemental to the other transcriptionally controlled stress response pathways (Schwechheimer and Kuehn, 2015). Thus osmotic stress has been suggested to influence the physiology of the *B. melitensis* 16M in biofilm, affecting the cell wall properties, and the composition of the envelope membrane and swarming capability of the cells is largely impaired.

As reported for other microorganisms (Hathroubi et al., 2018), the *B. melitensis* 16M biofilm cells have altered metabolism, typically thought to be associated with the restricted availability of nutrients. The expression of multiple genes involved in metabolism and translation, including nitric-oxide reductase large subunit, cytochrome *c*, and CbbQ/NirQ/NorQ/GpvN family protein, was downregulated in the *B. melitensis* 16M biofilm cells. This low energy metabolism biofilm state may be more conducive to the survival of *B*. *melitensis* 16M under osmotic conditions.

Gebetsberger et al. (2012) sequenced sRNAs co-purified with ribosomes of Haloferax volcanii, a halophilic archaeon, and identified multiple 5’ tRFs. They found that cells grown under elevated pH have abundant 5’ tRNA^Val^ fragment, which binds to small ribosomal subunits to inhibit translation globally. Several studies have also demonstrated that environmental stress increases cytosolic tRNA halves in microbes (Thompson et al., 2008; Garcia-Silva et al., 2010; Fricker et al., 2019; Raad et al., 2021). In our study, compared with the control group, there was increased tRNA transcription in the osmotic pressure-treated biofilm cells, which may be a self-regulatory mechanism of *B. melitensis* 16M to cope with the cell damage caused by the osmotic stress change. Taken together, these initial reports describing the ubiquity and the function of tRNA fragments in microbial cells and the differential expression of specific tRNA fragments suggest their possible roles in bacterial homeostasis and in regulating the expression of virulence factors.

The *B. melitensis* 16M biofilm cells may not actively block the translational machinery, as suggested by the upregulation of the gene encoding the 50s ribosomal protein under osmotic stress. This protein was previously described as being involved in prokaryotic translation with the 30s ribosomal protein. Unlike our study, Hathroubi et al. (2018) showed that transcription of RsfS within biofilms might act as a transcriptional repressor to maintain quiescent bacterial growth within *Helicobacter pylori* biofilms. The explanation for this may be that although the *Brucella* biofilm is destroyed by osmotic pressure, there are still active bacteria maintaining the growth of the biofilm.

It is possible to counterbalance the osmotic difference by accumulating compatible solutes, such as polyols and derivatives, sugars and derivatives, amino acids and derivatives, and betaines (Lamosa et al., 1998; Paul, 2013; Vyrides and Stuckey, 2017). Both *E. coli* and *B. subtilis* are known to accumulate and synthesize more compatible solutes than they can synthesize independently (Paul, 2013). Thus, physiological protection from stress is likely attributed to the stabilizing effect of compatible solutes on macromolecules and biosynthesis processes. The ABC transporter family involved in the transportation of compatible solutes functions as a high-affinity compatible solutes uptake system, which has the most significant protective effect on its growth when exposed to high salinity (Fraser et al., 2000). *Brucella* ABC transporters are responsible for nutrient uptake and the export of toxins and antibiotics (Ko and Splitter, 2000; Danese et al., 2004) and are required for *B. abortus* pathogenesis in the murine model (Rosinha et al., 2002). Considering that the chronicity of the infection with intracellular bacteria results from the combined effect of metabolic adaptations of the bacteria, ABC transporters which include the solutes transport system, play a central role in various metabolic and osmotic stress regulation pathways in bacteria. We speculate that the *Brucella* ABC transport system may be involved in regulating osmotic pressure response in biofilms. Osmotic stress alters the patterns of regulation of the DAGs involved in binding and transporting proteins. Changes in metabolic levels of these genes may aid in the acclimation and adaptation process to extracellular osmotic stress. The intracellular substances that protect the *B*. *melitensis* 16M biofilm cells under osmotic stress against osmosis include a variety of amino acids and ions and glycine betaine. We observed that the DAGs belonging to the ABC and glycine betaine transporters were significantly upregulated under osmotic stress. The OpuC ABC transporter family, the expression of which is induced by salt stress, is an ABC transporter family involved in the transportation of compatible solutes that function as a high-affinity glycine betaine uptake system (Hoffmann et al., 2013). To gain insight into the osmotic adaptative processes of ABC transporters in the biofilm, we chose the BME_RS12880 genes, which are upregulated in RNA-seq and significantly upregulated in real-time PCR of the 12 ABC transporters for further analysis. BME_RS12880 is predicted to be the substrate-binding domain of the ABC-type transporter OpuA, which has a high affinity multicomponent binding-protein-dependent transport system specific to betaine compounds for osmoregulation. The proteins are typically comprised of two globular subdomains connected by a flexible hinge and bind their ligand in the cleft between these domains resembling a Venus flytrap. The BME_RS12880 protein is predicted to have high affinity for betaine by molecular docking, suggesting that BME_RS12880 may respond to osmotic stress by accumulating betaine, however, further experiments are needed to verify the binding activity. When bacteria or the biofilm was exposed to 1.5 M NaCl, betaine had a significant protective effect on its growth. Biofilms formed by mutants of BME_RS12880 show growth defects and reduced biomass. Moreover, the high expression level of BME_RS12880 seems to be able to rescue the growth defects of the ΔBME_RS12880 strain under osmotic stress conditions.

In conclusion, the *B. melitensis* 16M biofilm under osmotic stress, which exhibited reduced clumps and biofilm formation, was still viable. Transcriptome sequence analysis revealed that the *B. melitensis* 16M biofilm cells could survive osmotic stress by upregulating or downregulating a series of stress response-related genes. Moreover, we identified one specific locus, BME_RS12880, a glycine betaine target that contributes directly to the development of osmotic tolerance in the *B. melitensis* 16M biofilm. We provide the key components in the molecular basis of *B. melitensis* 16M biofilm salt adaptation.

## Data availability statement

The data presented in this study are deposited in the Sequence Read Archive (SRA) repository, accession number PRJNA859018.

## Author contributions

LL and HZ conceived and designed the experiments. JG participated in the experiments and wrote the manuscript. JG, JZ, TZ, ZS, SS, YZ, DZ, SC, XD, YC, YS, SM, and CC performed the experiments. JG collected the experimental materials. All authors read and approved the final manuscript.

## Footnotes

1
https://www.ncbi.nlm.nih.gov/Structure/cdd/wrpsb.cgi

2
https://string-db.org/cgi/input.pl

3
http://www.sbg.bio.ic.ac.uk/phyre2/html/page.cgi?id=index

4
https://Pubchem.ncbi.nlm.nih.gov/

## Supplementary material

Supplementary Figure 1 Growth curve of *B. melitensis* 16M under osmotic stress. **(A)** Growth of the *B. melitensis* 16M strain in Brucella broth under NaCl, KCl, sucrose, and dextran. **(B)** Growth of the *B. melitensis* 16M strain in Brucella broth under 0.4, 0.8, 1.5, 2.5 M NaCl. Error bars represent the standard deviations of three independent experiments.

Supplementary Figure 2 Quantitative analysis of the biofilm by crystal violet staining. **(A,B)** Biofilm growth of *B. melitensis* 16M in the presence of the 1.5 M NaCl or not for 20 days and the biomass was quantified with 0.1% CV. Number 1–10 stand for represents ten independent tubes. Error bars represent standard error (*n* ≥ 3). ^**^*P* ≤ 0.01, unpaired Student’s *t*-test.

Supplementary Figure 3 Morphological observation and quantitative analysis of outer membrane vesicles. **(A)** Transmission electron microscopy (TEM) images of pure OMV from *B. melitensis* 16M biofilms under 1.5 M NaCl and control conditions. Scale bars = 200 nm. **(B)** Purified OMVs were quantified using nanoparticle tracking. Error bars represent standard error (*n* ≥ 3). ^**^*P* ≤ 0.01, unpaired Student’s *t*-test.

Supplementary Figure 4 Comparative analysis of multilayered three-and two-dimensional biofilms under osmotic stress. **(A)** Live confocal imaging of stacks of the *B. melitensis* 16M grown in 1.5 M NaCl or control conditions on a three-dimensional scaffold. Scale bars = 20 μm. Confocal images were subjected to quantitative analysis using the Comstat2 program to determine the biofilm biomass **(B)** and the average and maximum biofilm thickness **(C)**. **(D)** Biomass of *B. melitensis* 16M was quantified with 0.1% CV. **(E)** Survival of biofilm cells after 1.5 M NaCl treatment or control conditions. Error bars represent standard error (*n* ≥ 3). ^**^*P* ≤ 0.01, unpaired Student’s *t*-test.

Supplementary Figure 5 Analysis of conserved domains of BME_RS12880 gene and protein interaction network. **(A)** Prediction of the functional domain of BME_RS12880 protein in *B. melitensis* 16M. **(B)** Construction of a protein-protein correlation network according to the prediction of potential targets using protein interaction network analysis.

Supplementary Figure 6 Growth curves of the wt, ΔBME_RS12880, and ΔBME_RS12880-C strain in Brucella broth under control conditions. Error bars represent the standard deviations of three independent experiments.

Supplementary Figure 7 Quantitative analysis of the aggregates by crystal violet staining and bacterial plate count. **(A)** The biofilm of wt, ΔBME_RS12880, and ΔBME_RS12880-C strain under osmotic stress or Supplemented with 1 mM betaine and quantified with 0.1% CV. **(B)** Survival of wt, ΔBME_RS12880 and ΔBME_RS12880-C strain in biofilms grown under osmotic stress or Supplemented with 1 mM betaine. Error bars represent standard error (*n* ≥ 3). ^**^*P* ≤ 0.01, unpaired Student’s *t*-test.

Supplementary Figure 8 Intracellular replication capability of wt, ΔBME_RS12880, and ΔBME_RS12880-C in RAW 264.7 macrophages. At 0, 4, 8, 12, 24, and 48 h after infection, infected macrophages were lyzed, and supernatants were diluted for CFU enumeration. Error bars represent standard error (*n* ≥ 3). ^**^*P* ≤ 0.01, unpaired Student’s *t*-test.
