# Supplementary material for: Survival characteristics and transcriptome profiling reveal the adaptive response of the Brucella melitensis 16M biofilm to osmotic stress
Source: Front Microbiol. 2022 Aug 17;13:968592. doi: 10.3389/fmicb.2022.968592 (PMC9428795; doi:10.3389/fmicb.2022.968592)
Supplement: Supplementary file 1 [file Table_1.DOC]

**Supplementary Table 1** Primers of qRT-PCR used for this work.

| Primer | Sequence |
| --- | --- |
| BME_RS06085-F | CTCCTGTATGATCCGCGTGC |
| BME_RS06085-R | CCGGCCATAGGTTGAATCGC |
| BME_RS12870-F | TCCTGTATGATCCGCGTGC |
| BME_RS12870-R | CGCGCCCATAGAGGAAGC |
| BME_RS13630-F | CTTGTCAAACGGCCCCAGC |
| BME_RS13630-R | TGCCACGACCAGATATCGA |
| BME_RS12880-F | GCAAACTAACCAGATTCGG |
| BME_RS12880-R | ACCAGCTCCACCTTCTGGC |
| BME_RS12875-F | ATTTCAGTGTCGGCGGATG |
| BME_RS12875-R | AAAACCGCAAATTTCCAGC |
| BME_RS01965-F | AAAAAACTATTGATCGCGC |
| BME_RS01965-R | GAACTGTTCCTGCTGAACC |
| BME_RS02190-F | TCCACTGGCCCGTTCAAGG |
| BME_RS02190-R | ACATCGGCGGTTACGGTCG |
| BME_RS06090-F | TGACGGGTGTATTGGGTGC |
| BME_RS06090-R | CGAAGATCGCCGAAGCTAC |
| BME_RS02065-F | CGCCAGCCGCCTTGTCATC |
| BME_RS02065-R | CCGCAAATCCCCGTGCCAG |
| BME_RS02355-F | ACGACTCGAAGCCTATCCG |
| BME_RS02355-R | GCGTGACTGGTTGGAAAGC |
| BME_RS02180-F | GTCTATCTGGTTCCGACCTTCA |
| BME_RS02180-R | CAGCAAGTTCCAGACATAAAGG |
| BME_RS11830-F | GTCGAAATGAAAGACGTGCGCC |
| BME_RS11830-R | CCCCTCGATATGAATCGAACCC |
| 16S rRNA-F | ACTAAGGGCGAGGGTTGC |
| 16S rRNA-R | CACTGGACCATTACTGACGC |
