# Supplementary material for: Survival characteristics and transcriptome profiling reveal the adaptive response of the Brucella melitensis 16M biofilm to osmotic stress
Source: Front Microbiol. 2022 Aug 17;13:968592. doi: 10.3389/fmicb.2022.968592 (PMC9428795; doi:10.3389/fmicb.2022.968592)
Supplement: Supplementary file 2 [file Table_2.DOC]

| **Primer** | **Sequence** |
| --- | --- |
| BME_RS12880-N-F | TTCCGGCGGCAATGTTTTTC |
| BME_RS12880-N-R | GACATTCATCCCAGGTGGCACCTGCTCCTTCTCCTTCTC |
| BME_RS12880-C-F | TCTGGGGTTCGAAATGACCGGGGAGCTTTTATCCTCGTCG |
| BME_RS12880-C-R | CTGCTTGCAAATATGGCGCC |
| *kan*-F | GCCACCTGGGATGAATGTC |
| *kan*-R | CGGTCATTTCGAACCCCAGA |
| BME_RS12880-F-C | TGAATTCATGTTGGGCAAACTAACCA |
| BME_RS12880-R-C | GAATTC TTATTGCGAAAGCCATTCCT |

**Supplementary Table 2** Primers of mutants and complementation used for this work.
