# Supplementary material for: Survival characteristics and transcriptome profiling reveal the adaptive response of the Brucella melitensis 16M biofilm to osmotic stress
Source: Front Microbiol. 2022 Aug 17;13:968592. doi: 10.3389/fmicb.2022.968592 (PMC9428795; doi:10.3389/fmicb.2022.968592)
Supplement: Supplementary file 3 [file Table_3.doc]

**Supplementary Table 3** Upregulated genes in *B. melitensis* 16M biofilm using RNA-seq analysis, grouped by functional role categories.

| **Locus** | **Putative identification** | **Fold change** |
| --- | --- | --- |
| **Flagellar assembly** | | |
| *flgL* (BME_RS10960) | flagellar hook-associated family protein | 5.3 |
| *flgE* (BME_RS10950) | flagellar hook protein FlgE | 6.1 |
| *flaF* (BME_RS10965) | flagellar biosynthesis regulator FlaF | 5.0 |
| *fliC* (BME_RS10910) | flagellin | 3.6 |
| *flbT* (BME_RS10970) | flagellar biosynthesis repressor FlbT | 4.4 |
| *flgD* (BME_RS10975) | flagellar hook assembly protein FlgD | 4.7 |
| *flgK* (BME_RS10980) | flagellar hook-associated protein FlgK | 5.6 |
| *fliQ* (BME_RS10980) | flagellar biosynthetic protein FliQ | 5.5 |
| **Cell envelope** | | |
| BME_RS01010 | YadA-like membrane anchor | 3.0 |
| BME_RS03560 | porin family protein | 2.3 |
| **sRNA regulation** | | |
| BME_RS02400 | aminoacyl-tRNA hydrolase | 2.0 |
| BME_RS07130 | tRNA-Gln | 2.5 |
| BME_RS00965 | tRNA-Met | 3.2 |
| BME_RS11400 | tRNA-Leu | 2.6 |
| BME_RS06195 | tRNA-Arg | 2.1 |
| BME_RS01935 | tRNA-Met | 5.0 |
| BME_RS10430 | tRNA-Val | 2.4 |
| BME_RS11085 | tRNA-Met | 2.0 |
| **Transport and binding proteins** | | |
| BME_RS06085 | amino acid ABC transporter permease | 3.2 |
| *proV* (BME_RS12870) | glycine betaine/L-proline ABC transporter ATP-binding protein ProV | 2.6 |
| BME_RS13630 | ABC transporter permease | 2.5 |
| BME_RS14690 | ABC transporter ATP-binding protein | 2.6 |
| BME_RS12880 | glycine betaine ABC transporter substrate-binding protein | 2.3 |
| BME_RS12875 | proline/glycine betaine ABC transporter permease | 2.6 |
| BME_RS08650 | ABC transporter ATP-binding protein | 3.2 |
| BME_RS03305 | calcium-binding protein | 2.4 |
| BME_RS01965 | substrate-binding domain-containing protein | 2.2 |
| BME_RS02190 | ABC transporter ATP-binding protein | 2.1 |
| BME_RS06090 | amino acid ABC transporter substrate-binding protein | 2.2 |
| BME_RS02065 | ABC transporter permease subunit | 2.1 |
| BME_RS02355 | ABC transporter ATP-binding protein | 2.2 |
| BME_RS14695 | ABC transporter substrate-binding protein | 2.1 |
| BME_RS02180 | ABC transporter permease subunit | 2.6 |
| BME_RS11830 | methionine ABC transporter ATP-binding protein | 2.1 |
| **Regulatory functions** | | |
| BME_RS02820 | DnaJ family molecular chaperone | 2 |
| BME_RS00720 | antibiotic biosynthesis monooxygenase | 2.3 |
| BME_RS02600 | cold-shock protein | 2.7 |
| **Cellular processes** | | |
| BME_RS13530 | BA14K family protein | 2.1 |
| BME_RS10985 | FHIPEP family type III secretion protein | 3.7 |
| **Translation** | | |
| BME_RS11540 | ribonuclease P protein component | 2.4 |
| *rplT* (BME_RS09935) | 50s ribosomal protein | 2.7 |
| *Ffh* (BME_RS01105) | signal recognition particle protein | 2.3 |
| *rpmD* (BME_RS03870) | 50s ribosomal protein | 2 |
| *rplS* (BME_RS00740) | 50s ribosomal protein | 2.2 |
| *rpsL* (BME_RS03755) | 30s ribosomal protein | 2.2 |
| *rpmI* (BME_RS09940) | 50s ribosomal protein | 2.5 |
| *rpmG* BME_RS13425 | 50s ribosomal protein | 2.6 |
| *rpmE* (BME_RS01560) | 50s ribosomal protein | 2.1 |
| BME_RS17505 | GNAT family N-acetyl transferase | 2.8 |
| BME_RS08275 | site-specific integrase | 2.1 |
| BME_RS01005 | GNAT family N-acetyl transferase | 3.3 |
| **Energy metabolism** | | |
| leuA (BME_RS02255) | 2-isopropylmalate synthase | 2.2 |
| BME_RS11700 | alpha/beta hydrolase | 2 |
| BME_RS07330 | Prenyl transferase | 2.2 |
| BME_RS14995 | lipase | 2.5 |
| BME_RS17665 | aminotransferase class III-fold pyridoxal phosphate-dependent enzyme | 2.3 |
| BME_RS14015 | homoserine O-succinyl transferase | 2.3 |
| BME_RS05120 | glutathione S-transferase | 2.2 |
| BME_RS13015 | alkyl hydroperoxide reductase AhpD | 2.2 |
| BME_RS03120 | PLP-dependent aminotransferase family protein | 2.3 |
| BME_RS05120 | glutathione S-transferase | 2.2 |
| **Hypothetical proteins** | | |
| BME_RS09930 | hypothetical protein | 3.0 |
| BME_RS03115 | hypothetical protein | 2.5 |
| BME_RS12425 | Protein of unknown function | 2.1 |
| BME_RS05825 | hypothetical protein | 2.2 |
| BME_RS12375 | Protein of unknown function | 2.1 |
| BME_RS07325 | hypothetical protein | 2.5 |
| BME_RS05410 | hypothetical protein | 2.6 |
| BME_RS00970 | hypothetical protein | 2.4 |
