# Supplementary material for: Survival characteristics and transcriptome profiling reveal the adaptive response of the Brucella melitensis 16M biofilm to osmotic stress
Source: Front Microbiol. 2022 Aug 17;13:968592. doi: 10.3389/fmicb.2022.968592 (PMC9428795; doi:10.3389/fmicb.2022.968592)
Supplement: Supplementary file 4 [file Table_4.doc]

**Supplementary Table 4** Downregulated genes in *B. melitensis* 16M biofilm using RNA-seq analysis, grouped by functional role categories.

| **Locus** | **Putative identification** | **Fold change** |
| --- | --- | --- |
| **Flagellar assembly** | | |
| *flgG* (BME_RS15475) | flagellar basal-body rod protein FlgG | -2.9 |
| BME_RS10925 | MotB family protein | -2.7 |
| *flgH* (BME_RS15455) | flagellar basal body L-ring protein FlgH | -2.7 |
| *flgI* (BME_RS15465) | flagellar basal body P-ring protein FlgI | -2.2 |
| *fliK* (BME_RS10935) | flagellar hook-length control protein FliK | -2.6 |
| **Cell envelope** | | |
| BME_RS02270 | OmpW family protein | -2.5 |
| BME_RS10345 | lipoprotein | -2.5 |
| BME_RS10370 | OmpA family protein | -2.2 |
| *exbD* (BME_RS01775) | TonB system transport protein ExbD | -2.2 |
| *exbB* (BME_RS01780) | tonB-system energizer ExbB | -2.8 |
| **sRNA regulation** | | |
| BME_RS02680 | tRNA-Ile | -2.7 |
| BME_RS07650 | tRNA1(Val) (adenine(37)-N6)-methyltransferase | -2.1 |
| **Transport and binding proteins** | | |
| BME_RS15065 | hemerythrin domain-containing protein | -8.1 |
| BME_RS14215 | MFS transporter | -4.5 |
| BME_RS14125 | HlyD family efflux transporter periplasmic adaptor subunit | -4 |
| BME_RS14115 | ABC transporter permease | -3.2 |
| BME_RS10610 | ABC transporter permease | -2.9 |
| *cydD* (BME_RS13920) | thiol reductant ABC exporter subunit CydD | -2.8 |
| BME_RS10615 | ABC transporter permease | -2.8 |
| BME_RS13055 | ABC transporter substrate-binding protein | -2.8 |
| BME_RS14775 | carbohydrate ABC transporter permease | -2.8 |
| BME_RS14905 | ABC transporter permease | -2.7 |
| BME_RS09210 | ABC transporter ATP-binding protein | -2.6 |
| BME_RS10805 | Tm-1-like ATP-binding domain-containing protein | -2.5 |
| BME_RS11860 | ABC transporter ATP-binding protein | -2.5 |
| BME_RS10660 | cation-translocating P-type ATPase | -2.5 |
| BME_RS09205 | ABC transporter permease | -2.5 |
| BME_RS10765 | ABC transporter ATP-binding protein | -2.4 |
| BME_RS08835 | sulfate transporter family protein | -2.4 |
| BME_RS08525 | sugar ABC transporter substrate-binding protein | -2.3 |
| BME_RS11005 | rod-binding protein | -2.3 |
| BME_RS06410 | LysE/ArgO family amino acid transporter | -2.3 |
| BME_RS10780 | ABC transporter substrate-binding protein | -2.3 |
| BME_RS07875 | TRAP transporter substrate-binding protein | -2.3 |
| BME_RS14865 | ABC transporter permease | -2.2 |
| BME_RS11845 | branched-chain amino acid ABC transporter permease | -2.1 |
| BME_RS08515 | carbohydrate ABC transporter permease | -2.1 |
| BME_RS08580 | ABC transporter ATP-binding protein | -2.1 |
| BME_RS13095 | carbohydrate ABC transporter permease | 2.0 |
| BME_RS13270 | branched-chain amino acid ABC transporter permease | 2.0 |
| *cydC* (BME_RS13915) | thiol reductant ABC exporter subunit CydC | -3.2 |
| BME_RS06265 | DMT family transporter | -2.7 |
| *ugpC* (BME_RS08510) | sn-glycerol-3-phosphate ABC transporter ATP-binding protein | -2.5 |
| BME_RS08520 | sugar ABC transporter permease | -2.4 |
| BME_RS04775 | binding protein | -2 |
| BME_RS11850 | branched-chain amino acid ABC transporter permease | -2.1 |
| **Regulatory functions** | | |
| BME_RS14130 | CerR family C-terminal domain-containing protein | -4.1 |
| BME_RS14925 | regulatory protein NosR | -3.8 |
| BME_RS10400 | Hsp20 family protein | -3.7 |
| BME_RS13925 | GbsR/MarR family transcriptional regulator | -3.3 |
| BME_RS10875 | helix-turn-helix domain-containing protein | -3.3 |
| BME_RS06505 | helix-turn-helix domain-containing protein | -3.1 |
| BME_RS15010 | metal-sulfur cluster assembly factor | -2.8 |
| BME_RS12780 | MarR family transcriptional regulator | -2.4 |
| BME_RS09290 | ImuA family protein | -2.4 |
| BME_RS09300 | error-prone DNA polymerase | -2.3 |
| BME_RS11385 | universal stress protein | -2.3 |
| BME_RS12200 | organic hydroperoxide resistance protein | -2.3 |
| BME_RS02010 | MarC family protein | -2.2 |
| BME_RS06935 | GreA/GreB family elongation factor | -2.2 |
| *putA* (BME_RS12945) | trifunctional transcriptional regulator | -2.2 |
| BME_RS15385 | YdcH family protein | -2.1 |
| *tldD* (BME_RS07350) | metalloprotease TldD | -2.1 |
| BME_RS12065 | GntR family transcriptional regulator | -2.1 |
| BME_RS09295 | DNA polymerase Y family protein | -2.4 |
| BME_RS06790 | DNA-packaging protein | -2.1 |
| BME_RS04675 | universal stress protein | -2.5 |
| BME_RS01880 | DNA-3-methyladenine glycosylase | -2.1 |
| **Cellular processes** | | |
| BME_RS10335 | P-type DNA transfer protein VirB5 | -3.3 |
| BME_RS10340 | type IV secretion system protein VirB6 | -3.3 |
| BME_RS10325 | type IV secretion system protein virB3 | -3.2 |
| BME_RS10350 | virB8 family protein | -2.7 |
| BME_RS10330 | VirB4 family type IV secretion system protein | -2.6 |
| BME_RS10355 | P-type conjugative transfer protein VirB9 | -2.5 |
| BME_RS10360 | type IV secretion system protein VirB10 | -2.5 |
| **Translation** | | |
| *rsfS* (BME_RS01035) | ribosome silencing factor | -2.2 |
| *ybeY* (BME_RS09775) | rRNA maturation RNase YbeY | -2.0 |
| *thpR* (BME_RS09225) | RNA ligase superfamily | -2.1 |
| **Energy metabolism** | | |
| BME_RS15040 | nitric-oxide reductase large subunit | -8.1 |
| BME_RS15045 | cytochrome *c* | -7.7 |
| BME_RS15035 | CbbQ/NirQ/NorQ/GpvN family protein | -6.6 |
| BME_RS01950 | PepSY domain-containing protein | -6.4 |
| BME_RS17765 | cytosine permease | -5.4 |
| BME_RS10655 | oxygen-independent coproporphyrinogen III oxidase | -5.3 |
| BME_RS15030 | nitric oxide reductase activation protein NorD | -4.8 |
| BME_RS13910 | cytochrome ubiquinol oxidase subunit I | -4.6 |
| BME_RS10380 | amino acid permease | -4.4 |
| BME_RS15055 | cytochrome *c* oxidase subunit 3 | -4.4 |
| BME_RS13905 | cytochrome d ubiquinol oxidase subunit II | -4.4 |
| BME_RS13900 | cytochrome bd-I oxidase subunit CydX | -4.1 |
| BME_RS15390 | cytochrome *c* family protein | -4 |
| BME_RS14920 | nitrous-oxide reductase | -4 |
| BME_RS16675 | Bro-N domain-containing protein | -3.6 |
| BME_RS15050 | cytochrome C oxidase subunit IV family protein | -3.5 |
| BME_RS14845 | SCP2 domain-containing protein | -3.3 |
| BME_RS11105 | lactate dehydrogenase | -3.3 |
| BME_RS10585 | 4'-phosphopantetheinyl transferase | -3.3 |
| BME_RS10320 | TrbC/VirB2 family protein | -3.2 |
| BME_RS14110 | cupin domain-containing protein | -3.2 |
| BME_RS14640 | glutamate decarboxylase | -3.2 |
| BME_RS10495 | VOC family protein | -3.2 |
| BME_RS10485 | SDR family oxidoreductase | -3 |
| BME_RS10315 | lytic transglycosylase domain-containing protein | -3 |
| BME_RS01765 | phosphodiester glycosidase family protein | -3 |
| BME_RS14840 | UbiD family decarboxylase | -3 |
| BME_RS01175 | usg protein | -2.9 |
| BME_RS01160 | zinc metalloprotease HtpX | -2.9 |
| BME_RS06765 | phage tail tape measure protein | -2.9 |
| BME_RS13770 | UDP-glucose 4-epimerase GalE | -2.9 |
| BME_RS15405 | cytochrome *b* | -2.9 |
| BME_RS10475 | pyruvate dehydrogenase complex dihydrolipoamide acetyltransferase enzyme | -2.9 |
| BME_RS14185 | acyl-CoA synthetase | -2.8 |
| BME_RS14850 | U32 family peptidase | -2.8 |
| BME_RS00105 | enoyl-CoA hydratase/isomerase family protein | -2.8 |
| BME_RS06750 | C40 family peptidase | -2.7 |
| BME_RS03665 | transglutaminase-like cysteine peptidase | -2.7 |
| BME_RS14855 | U32 family peptidase | -2.7 |
| BME_RS13765 | glycosyltransferase | -2.7 |
| BME_RS05565 | glycoside hydrolase family 108 protein | -2.7 |
| BME_RS08620 | thiazole synthase | -2.7 |
| BME_RS00135 | GFA family protein | -2.7 |
| BME_RS00120 | 3-ketoacyl-ACP reductase | -2.7 |
| BME_RS00875 | glutaredoxin 3 | -2.6 |
| BME_RS02950 | SCO family protein | -2.6 |
| BME_RS14245 | class I SAM-dependent methyltransferase | -2.5 |
| BME_RS14915 | nitrous oxide reductase family maturation protein NosD | -2.5 |
| BME_RS14190 | 3-hydroxyacyl-CoA dehydrogenase | -2.5 |
| BME_RS15355 | glycolate oxidase subunit GlcE | -2.5 |
| BME_RS14260 | SDR family oxidoreductase | -2.5 |
| BME_RS04105 | SgcJ/EcaC family oxidoreductase | -2.5 |
| BME_RS01450 | YdcH family protein | -2.5 |
| BME_RS13310 | 4-hydroxybenzoate 3-monooxygenase | -2.5 |
| BME_RS08760 | phosphoadenylyl-sulfate reductase | -2.5 |
| BME_RS14425 | Gfo/Idh/MocA family oxidoreductase | -2.5 |
| BME_RS10470 | shikimate dehydrogenase | -2.5 |
| BME_RS08555 | sarcosine oxidase subunit beta family protein | -2.5 |
| BME_RS17525 | GMC family oxidoreductase N-terminal domain-containing protein | -2.5 |
| BME_RS01885 | HNH endonuclease | -2.5 |
| BME_RS10130 | ATP-dependent protease subunit HslV | -2.4 |
| BME_RS09400 | SH3 domain-containing protein | -2.4 |
| BME_RS08775 | siroheme synthase CysG | -2.4 |
| BME_RS14990 | copper-containing nitrite reductase | -2.4 |
| BME_RS15160 | enoyl-CoA hydratase | -2.4 |
| BME_RS13760 | UDP-glucose/GDP-mannose dehydrogenase family protein | -2.4 |
| BME_RS11135 | aspartate aminotransferase family protein | -2.4 |
| BME_RS14635 | amino acid permease | -2.4 |
| BME_RS03295 | SDR family oxidoreductase | -2.4 |
| BME_RS09530 | methylcrotonoyl-CoA carboxylase | -2.4 |
| BME_RS14205 | peptide-methionine (R)-S-oxide reductase MsrB | -2.4 |
| BME_RS14195 | acetyl-CoA C-acyltransferase | -2.4 |
| BME_RS14835 | UbiX family flavin prenyltransferase | -2.4 |
| BME_RS03680 | CBS domain-containing protein | -2.4 |
| BME_RS00870 | ComF family protein | -2.4 |
| BME_RS14200 | acyl-CoA dehydrogenase family protein | -2.4 |
| BME_RS14745 | glutaredoxin-like protein NrdH | -2.4 |
| BME_RS00925 | ATP-dependent chaperone ClpB | -2.3 |
| BME_RS00695 | tyrosine recombinase XerC | -2.3 |
| BME_RS09000 | ATP-dependent helicase HrpB | -2.3 |
| BME_RS08545 | sarcosine oxidase subunit alpha | -2.3 |
| BME_RS06605 | sel1 repeat family protein | -2.3 |
| BME_RS09865 | transglutaminase-like cysteine peptidase | -2.3 |
| BME_RS14505 | phosphoketolase family protein | -2.3 |
| BME_RS08485 | SDR family oxidoreductase | -2.3 |
| BME_RS08855 | DUF1150 family protein | -2.3 |
| BME_RS14490 | cytochrome P450 | -2.3 |
| BME_RS07600 | DUF6101 family protein | -2.2 |
| BME_RS08610 | glycine oxidase ThiO | -2.2 |
| BME_RS10460 | magnesium-translocating P-type ATPase | -2.2 |
| BME_RS12140 | group III truncated hemoglobin | -2.2 |
| BME_RS12295 | carbohydrate kinase | -2.2 |
| BME_RS06975 | xylulokinase | -2.2 |
| BME_RS03090 | STAS/SEC14 domain-containing protein | -2.1 |
| BME_RS10365 | P-type DNA transfer ATPase VirB11 | -2.1 |
| BME_RS12010 | cupin domain-containing protein | -2.1 |
| BME_RS09525 | isovaleryl-CoA dehydrogenase | -2.1 |
| BME_RS12160 | thioredoxin | -2.1 |
| BME_RS08675 | GAF domain-containing protein | -2.1 |
| BME_RS10835 | 5-carboxymethyl-2-hydroxymuconate Delta-isomerase | -2.1 |
| BME_RS15425 | CoA transferase | -2 |
| BME_RS14045 | NAD(P)/FAD-dependent oxidoreductase | -2 |
| BME_RS11025 | transcriptional repressor | -2 |
| BME_RS05555 | sugar kinase | -2 |
| BME_RS09240 | DUF1232 domain-containing protein | -2 |
| BME_RS05980 | riboflavin synthase | -2 |
| BME_RS12995 | inositol 2-dehydrogenase | -2 |
| BME_RS15155 | 3-hydroxyacyl-CoA dehydrogenase | -2 |
| **Hypothetical proteins** | | |
| BME_RS17875 | hypothetical protein | -8.13 |
| BME_RS15020 | hypothetical protein | -4.8 |
| BME_RS17670 | hypothetical protein | -4.2 |
| BME_RS09215 | hypothetical protein | -3.5 |
| BME_RS11000 | hypothetical protein | -3.3 |
| BME_RS04950 | hypothetical protein | -2.5 |
| BME_RS11720 | hypothetical protein | -2.5 |
| BME_RS13740 | hypothetical protein | -2.4 |
| BME_RS07965 | hypothetical protein | -2.3 |
| BME_RS06405 | hypothetical protein | -2.3 |
| BME_RS13375 | hypothetical protein | -2.1 |
| BME_RS17140 | hypothetical protein | -2.1 |
| BME_RS15910 | hypothetical protein | -2.1 |
| BME_RS17810 | hypothetical protein | -2.0 |
| BME_RS15025 | Uncharacterized conserved protein | -7.5 |
| BME_RS15015 | Uncharacterized conserved protein | -3.3 |
| BME_RS15595 | hypothetical protein | -3.1 |
| BME_RS15565 | hypothetical protein | -2.4 |
| BME_RS14830 | Protein of unknown function | -2.7 |
| BME_RS13175 | Protein of unknown function | -2.6 |
| BME_RS14255 | Protein of unknown function | -2.6 |
| BME_RS08770 | Protein of unknown function | -2.3 |
| BME_RS07490 | Protein of unknown function | -2.3 |
