# Supplementary figures and images for: Survival characteristics and transcriptome profiling reveal the adaptive response of the Brucella melitensis 16M biofilm to osmotic stress
Source: Front Microbiol. 2022 Aug 17;13:968592. doi: 10.3389/fmicb.2022.968592 (PMC9428795; doi:10.3389/fmicb.2022.968592)

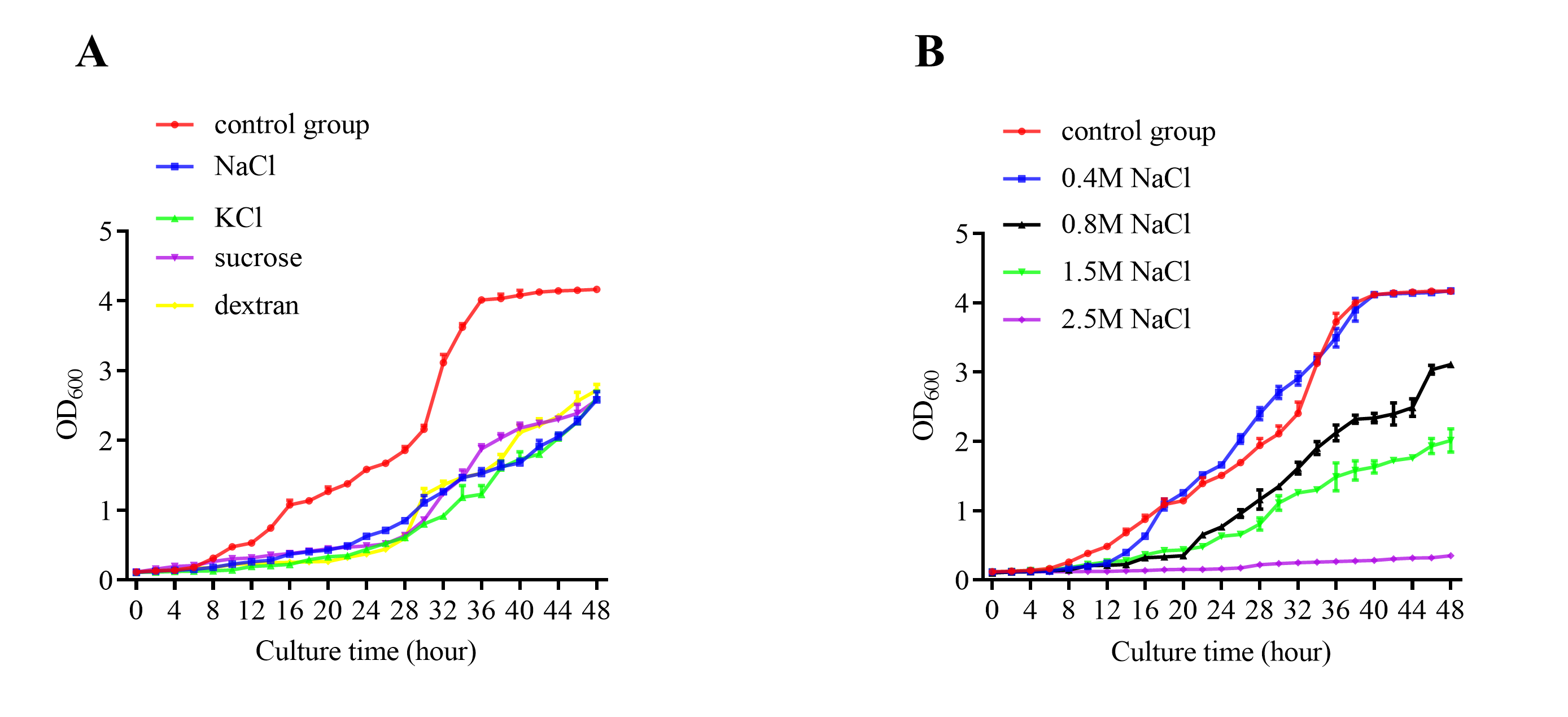

Supplement: Supplementary Figure 1 — Growth curve of B. melitensis 16M under osmotic stress. (A) Growth of the B. melitensis 16M strain in Brucella broth under NaCl, KCl, sucrose, and dextran. (B) Growth of the B. melitensis 16M strain in Brucella broth under 0.4, 0.8, 1.5, 2.5 M NaCl. Error bars represent the standard deviations of three independent experiments. [file Image_1.TIF]

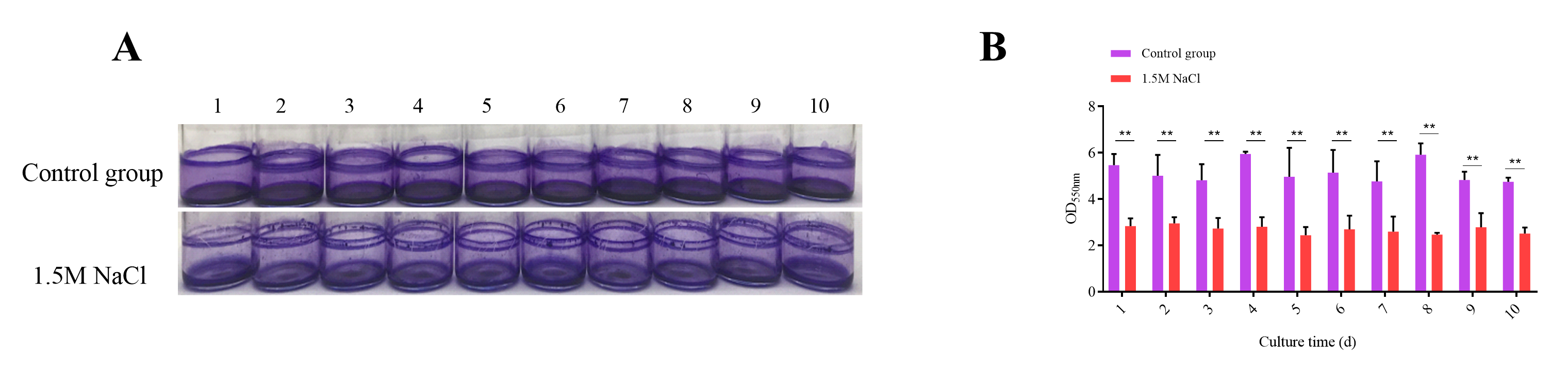

Supplement: Supplementary Figure 2 — Quantitative analysis of the biofilm by crystal violet staining. (A,B) Biofilm growth of B. melitensis 16M in the presence of the 1.5 M NaCl or not for 20 days and the biomass was quantified with 0.1% CV. Number 1–10 stand for represents ten independent tubes. Error bars represent standard error (n ≥ 3). **P ≤ 0.01, unpaired Student’s t-test. [file Image_2.TIF]

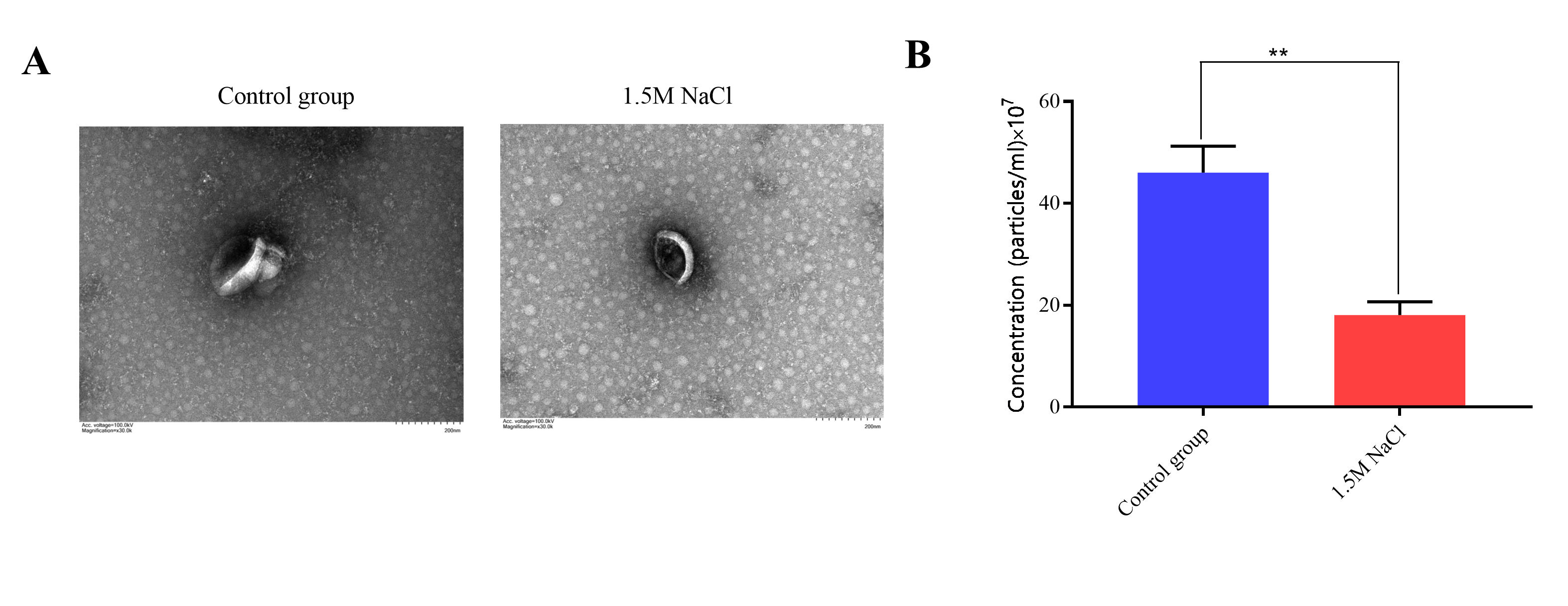

Supplement: Supplementary Figure 3 — Morphological observation and quantitative analysis of outer membrane vesicles. (A) Transmission electron microscopy (TEM) images of pure OMV from B. melitensis 16M biofilms under 1.5 M NaCl and control conditions. Scale bars = 200 nm. (B) Purified OMVs were quantified using nanoparticle tracking. Error bars represent standard error (n ≥ 3). **P ≤ 0.01, unpaired Student’s t-test. [file Image_3.TIF]

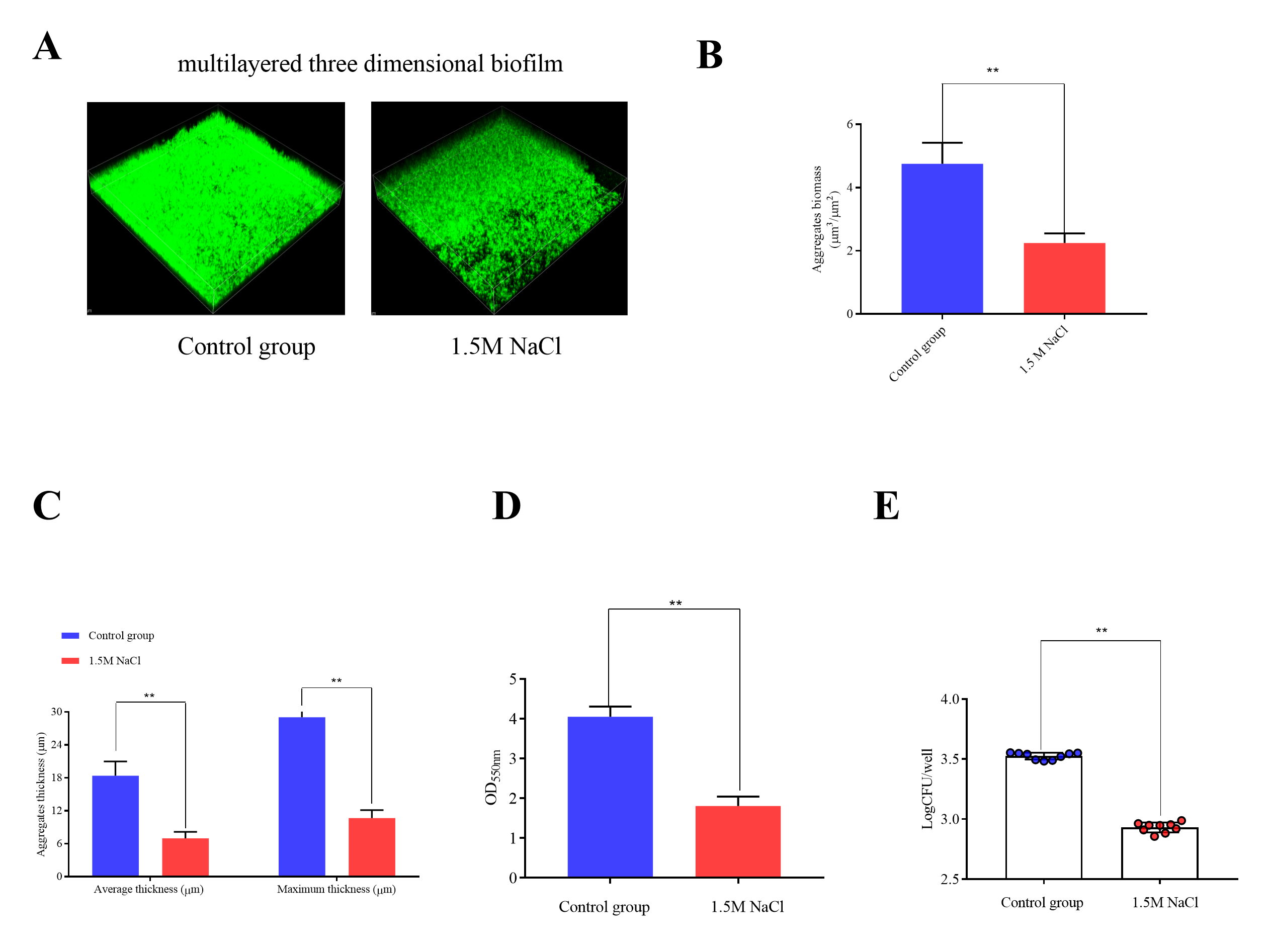

Supplement: Supplementary Figure 4 — Comparative analysis of multilayered three-and two-dimensional biofilms under osmotic stress. (A) Live confocal imaging of stacks of the B. melitensis 16M grown in 1.5 M NaCl or control conditions on a three-dimensional scaffold. Scale bars = 20 μm. Confocal images were subjected to quantitative analysis using the Comstat2 program to determine the biofilm biomass (B) and the average and maximum biofilm thickness (C). (D) Biomass of B. melitensis 16M was quantified with 0.1% CV. (E) Survival of biofilm cells after 1.5 M NaCl treatment or control conditions. Error bars represent standard error (n ≥ 3). **P ≤ 0.01, unpaired Student’s t-test. [file Image_4.TIF]

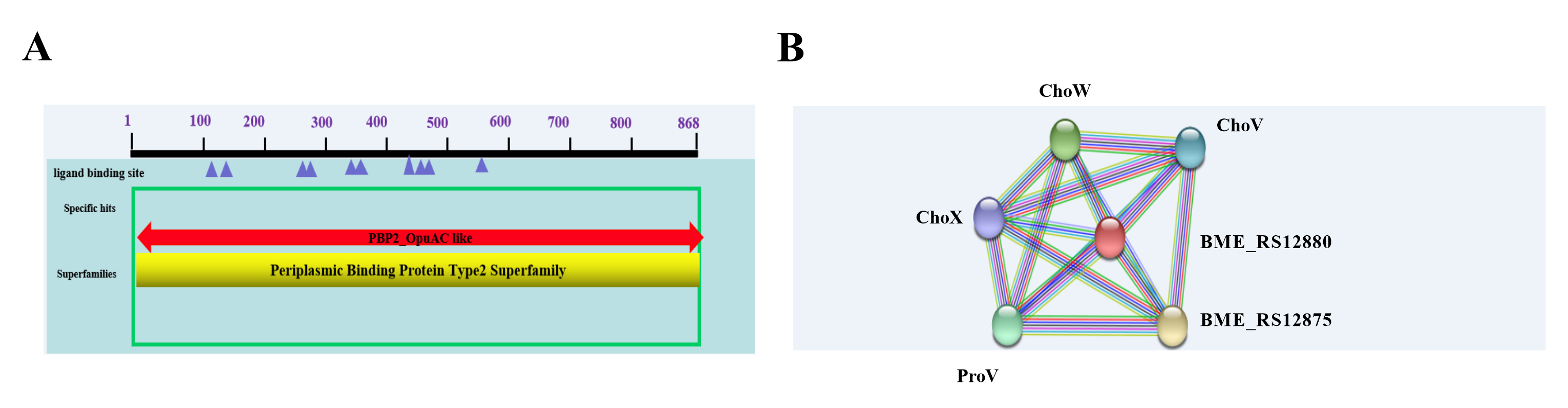

Supplement: Supplementary Figure 5 — Analysis of conserved domains of BME_RS12880 gene and protein interaction network. (A) Prediction of the functional domain of BME_RS12880 protein in B. melitensis 16M. (B) Construction of a protein-protein correlation network according to the prediction of potential targets using protein interaction network analysis. [file Image_5.TIF]

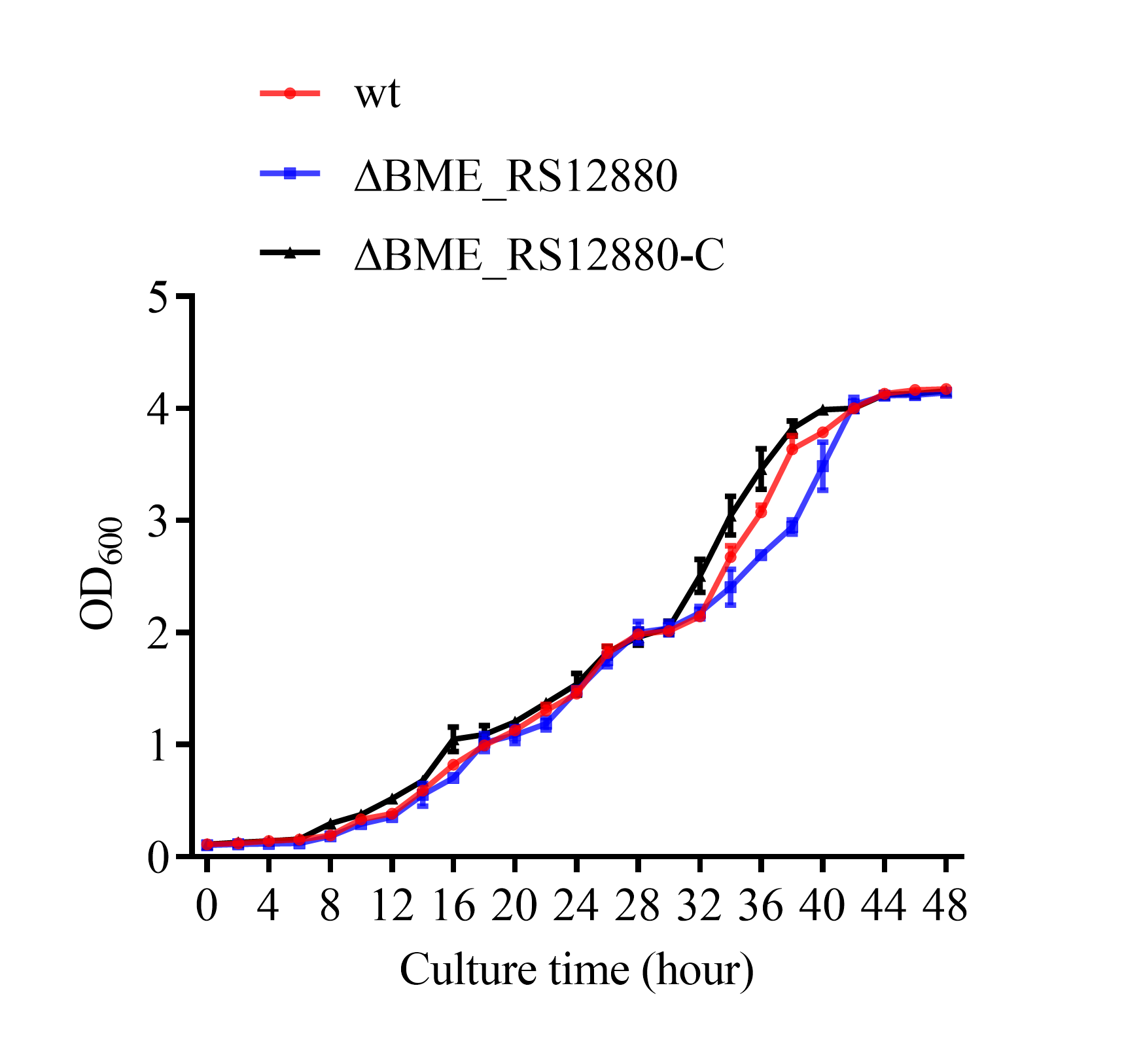

Supplement: Supplementary Figure 6 — Growth curves of the wt, ΔBME_RS12880, and ΔBME_RS12880-C strain in Brucella broth under control conditions. Error bars represent the standard deviations of three independent experiments. [file Image_6.TIF]

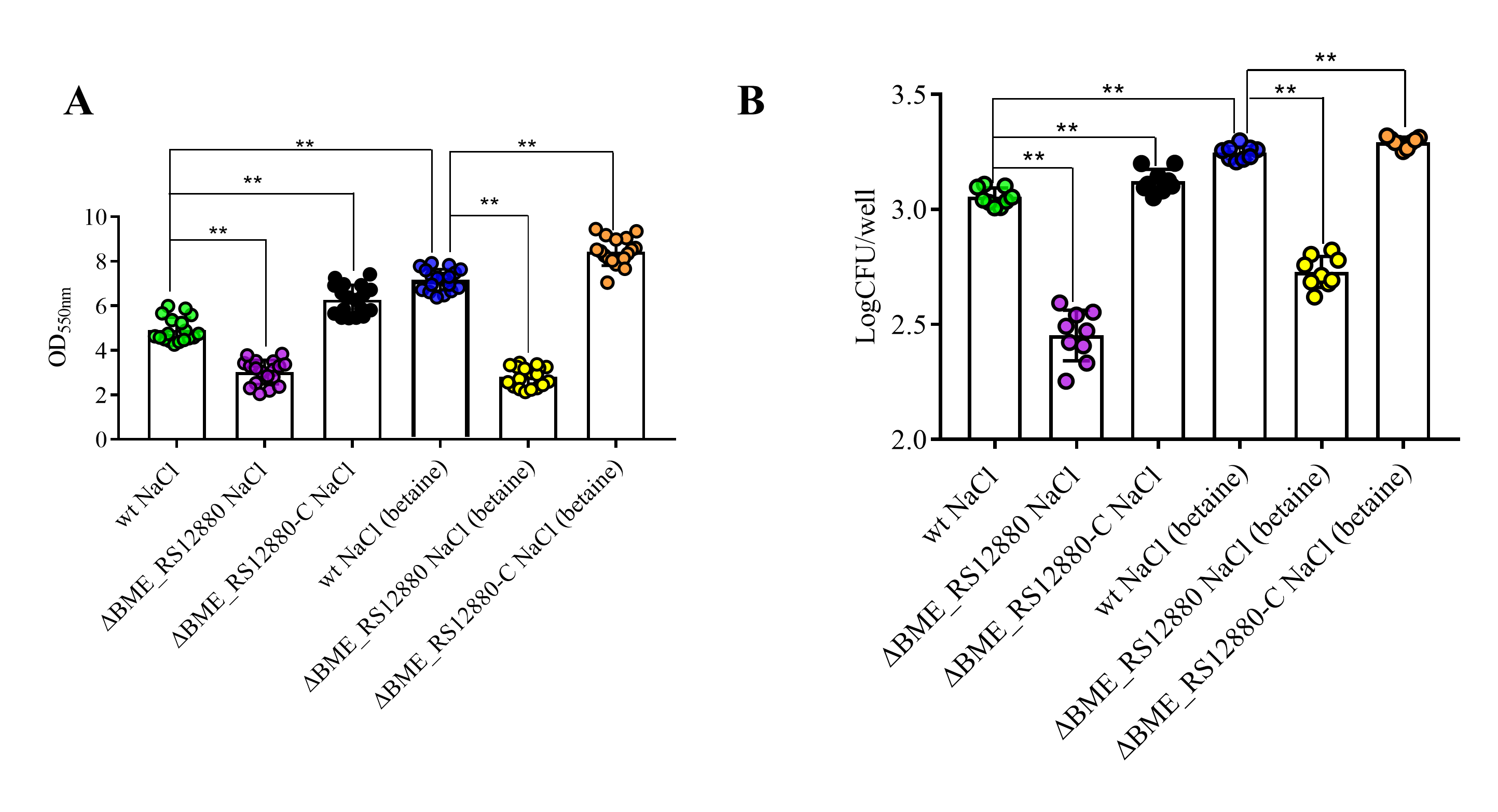

Supplement: Supplementary Figure 7 — Quantitative analysis of the aggregates by crystal violet staining and bacterial plate count. (A) The biofilm of wt, ΔBME_RS12880, and ΔBME_RS12880-C strain under osmotic stress or Supplemented with 1 mM betaine and quantified with 0.1% CV. (B) Survival of wt, ΔBME_RS12880 and ΔBME_RS12880-C strain in biofilms grown under osmotic stress or Supplemented with 1 mM betaine. Error bars represent standard error (n ≥ 3). **P ≤ 0.01, unpaired Student’s t-test. [file Image_7.TIF]

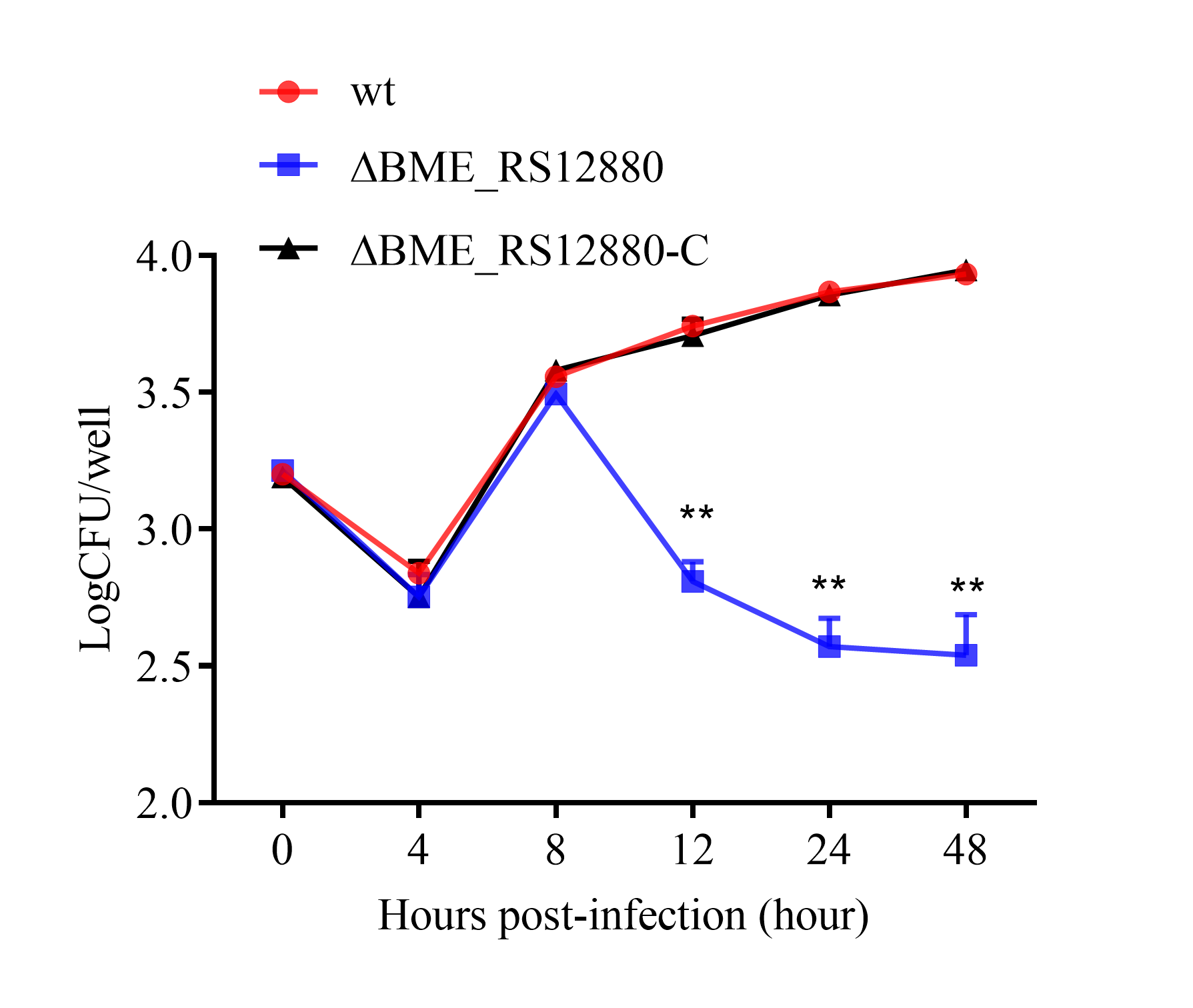

Supplement: Supplementary Figure 8 — Intracellular replication capability of wt, ΔBME_RS12880, and ΔBME_RS12880-C in RAW 264.7 macrophages. At 0, 4, 8, 12, 24, and 48 h after infection, infected macrophages were lyzed, and supernatants were diluted for CFU enumeration. Error bars represent standard error (n ≥ 3). **P ≤ 0.01, unpaired Student’s t-test. [file Image_8.TIF]
